# Maternal critical care infrastructure and organisational preparedness in Türkiye: a national analysis of the MaCriCare cross-sectional survey

**DOI:** 10.1080/07853890.2026.2742563

**Published:** 2026-10-10

**Authors:** Dudu Berrin Günaydın, Selin Erel, Miray Gözde Özdemir, Çiğdem Naz Ünver, Paweł Krawczyk, Steve Cantellow, Dominika Dabrowska

**Affiliations:** ^a^ Department of Anesthesiology and Reanimation, Faculty of Medicine, Gazi University, Ankara, Türkiye; ^b^ Department of Anesthesiology and Intensive Care, Jagiellonian University Medical College, Kraków, Poland; ^c^ Nottingham University Hospitals NHS Trust, Nottingham, UK; ^d^ Department of Anaesthetics and Intensive Care, West Middlesex University Hospital, Chelsea and Westminster NHS Foundation Trust, London, UK

**Keywords:** Critical care, intensive care units, Health services accessibility, pregnancy complications, surveys and questionnaires

## Abstract

**Objectives:**

MaCriCare (Maternal Critical Care) study, created as a European initiative, evaluated the availability of maternal critical care services. Since unique strengths and challenges of Turkish data within the MaCriCare study have not yet been evaluated separately, we aimed to assess maternal critical care preparedness and organisation in Turkish obstetric units participating in the MaCriCare cohort.

**Methods:**

This secondary analysis included validated unit responses collected from September 2021 to January 2022. Survey domians including access to an intensive care unit (ICU), modified early-obstetric warning-score (MEOWS) use, availability of critical-care resources, care locations for pre-eclampsia, postpartum haemorrhage (PPH) and sepsis, and maternal/staff support by focusing service accessibility and annual delivery rate were used. The comparator comprised 993 non-Türkiye units, and 140 Turkish units from 1,133-unit MaCriCare cohort.

**Results:**

Responses from 140/403 eligible units (34.7%) represented 305,250 annual deliveries where 501–1,500 and 1,501–3,000 annual deliveries comprised 32.1% and 30.0%, respectively. Rate of same-hospital ICU access was 78.6% and 82.4% and use of any MEOWS system was 27.9% and versus 35.4% in Turkish vs non-Türkiye units, respectively. For pre-eclampsia, 58/140 units (41.4%) reported obstetric-unit care before delivery, followed by separate critical care particularly for PPH varied between 62.1% to 82.9%. Sepsis rate was 86.4%. Unavailable advanced resources included cardiac output monitoring (30.0%), cell salvage (47.1%), and viscoelastic testing (67.1%). Rate of breastfeeding support was 92.7% and maternal psychological screening was 24.5%.

**Conclusions:**

Describing maternal critical-care infrastructure and reported organisational arrangements among participating Turkish units may support future service assessment and planning.

## Introduction

Severe maternal morbidity may require rapid access to critical care, advanced monitoring, blood products, diagnostic imaging and multidisciplinary services [1,2]. How these resources are organised and where they are located are therefore important components of maternity-service preparedness.

Maternal critical care (MaCriCare) was a cross-sectional observational study conducted across 26 countries in the World Health Organization (WHO) European Region (ER) using a structured survey to assess obstetric and critical-care services. The first three reports examined preparedness and reported care pathways for severe maternal morbidity, the availability and placement of clinical services and resources, and provisions relating to maternal experience, follow-up and staff support [1–3]. Türkiye contributed 140 of the 1,133 participating obstetric units (12.4%).

In Türkiye, 98.0% of births occurred in health institutions in 2020, with 1,112,859 live births recorded nationally [4]. Maternity care is provided through the Ministry of Health, university and private-sector hospitals, which differ in level of care, teaching status, annual delivery volume and access to critical-care services. The national caesarean-delivery rate was 59.6% in 2020, meaning that more than half of births were delivered by caesarean section [5]. This national context is relevant to the organisation of services for obstetric haemorrhage, anaesthesia, perioperative monitoring, blood-product support and maternal critical care.

To our knowledge, no previous national unit-level survey has specifically described maternal critical-care infrastructure across Türkiye. The present study is a secondary analysis of the Turkish MaCriCare responses. We aimed to describe maternal critical-care organisation in the participating Turkish obstetric units, including same-hospital ICU access, early-warning systems, clinical services and resources, reported care locations, and maternal and staff support. Findings were also examined according to unit characteristics and annual delivery volume and compared descriptively with the remaining 993 non-Türkiye MaCriCare units.

## Methods

### Participants and survey administration

The Turkish national coordinator identified 403 eligible obstetric units using the Turkish Society of Anaesthesiology and Reanimation’s database and obtained their contact details from the booklet, including center list of Turkish Anaesthesiology and Reanimation in the country and distributed the survey invitation *via* e-mail.

The survey was administered through LimeSurvey from September 2021 to January 2022. The MaCriCare Steering Committee developed the instrument using the brief, relevant, unambiguous, specific and objective (BRUSO) approach. Translation, piloting and national coordination followed the procedures reported for the original MaCriCare study [1,6]. Unfinished surveys received reminders at 30-day intervals. Only completed and validated responses were included. When duplicate completed responses were identified, the national coordinators contacted the unit to determine which record should be retained.

### Survey domains and definitions

The survey characterised participating obstetric units according to ownership, level of care, university and teaching status, annual delivery volume, access to an intensive care unit (ICU), and use of an obstetric early-warning system. Maternal critical-care capacity was evaluated through 31 clinical services and resources covering advanced monitoring; advanced cardiorespiratory and renal therapies; pharmacological therapies; access to blood products and cell salvage; point-of-care testing; diagnostic imaging; and other specialist clinical and diagnostic services. The survey also examined reported organisational arrangements for four standardised severe-maternal-morbidity scenarios and seven provisions relating to maternal experience, follow-up and staff support [1–3].

Annual delivery volume was self-reported using seven predefined categories: 1–500, 501–1,500, 1,501–3,000, 3,001–4,500, 4,501–6,000, 6,001–7,500 and >7,500 deliveries per year. Same-hospital ICU access was defined as access to an ICU within the same institution as the obstetric unit.

Following the reporting convention used in the parent MaCriCare study, early-warning-system use was analysed under a broad MEOWS category [1]. The questionnaire asked whether units used a modified early obstetric warning score (MEOWS) or another early-warning system, with ‘Yes’, ‘No’ and ‘Other’ response options. This grouping describes reported system use and does not imply that the different tools are clinically equivalent.

The four clinical scenarios were pre-eclampsia, stable major postpartum haemorrhage (PPH), unstable major PPH and sepsis. For pre-eclampsia, the response options distinguished between care in the obstetric unit before and after delivery; care in the obstetric unit before delivery followed by separate critical care; separate critical care before and after delivery; transfer to another hospital; and ‘Other’. For stable PPH, unstable PPH and sepsis, responses identified whether care was usually provided entirely within the obstetric unit, in separate critical care, through transfer to another hospital, or under another arrangement. Responses were mutually exclusive within each scenario and described the unit’s usual reported care location rather than patient-level management. The questionnaire did not collect the reasons for transfer or non-transfer.

For the 31 clinical services and resources, respondents indicated whether each was located in the same ward or an adjoining area, the same building, a different building, or a different hospital, or was not available.

The seven maternal and staff support provisions comprised breastfeeding support or initiation; physical mother–baby contact; virtual mother–baby contact; separate accommodation for maternity patients; post-discharge follow-up in a critical-care clinic or equivalent service; maternal psychological screening or support; and staff debriefing or psychological support following a poor maternal outcome. These items were assessed among units reporting same-hospital ICU access.

### Statistical analysis

Data were analysed using Stata version 17 (StataCorp LLC, College Station, TX, USA). Categorical variables are presented as *n*/*N* (%), where *n* is the number of units in the reported category and *N* is the number of units in the corresponding group. Two-sided 95% confidence intervals were calculated using the Clopper–Pearson exact binomial method for the overall Turkish proportions reporting same-hospital ICU access and use of MEOWS or another early-warning system. These intervals describe uncertainty under a binomial model; they do not account for selective nonresponse or establish national representativeness. Comparisons across annual delivery-volume categories and between Turkish and non-Türkiye units were descriptive, and no hypothesis tests were performed. For Turkish strata containing fewer than 10 units, results are presented as *n*/*N* without percentages. No a priori sample-size or power calculation was performed because this secondary analysis included all validated Turkish MaCriCare responses.

The non-Türkiye comparator comprised 993 units, derived by subtracting the 140 Turkish units from the full 1,133-unit MaCriCare cohort. For the maternal experience, follow-up and staff-support items, the corresponding non-Türkiye comparator comprised 818 units, derived by subtracting the 110 eligible Turkish units from the 928-unit MaCriCare support module. ‘Other’ responses were retained as a separate category where applicable.

Survey coverage was assessed according to both the proportion of eligible obstetric units that responded and the approximate proportion of national births represented by the participating units. Because annual delivery volume was reported in categories, the midpoint of each category was assigned to each unit to estimate its annual deliveries. For the >7,500 category, 8,750 deliveries were assigned as the midpoint between the lower boundary of 7,500 and the assumed maximum of 10,000 used in the full MaCriCare analysis [1].

### Ethical approval

The original Turkish MaCriCare study was approved by the Gazi University Ethics Committee (approval no. 188, 22 February 2021). The use of the Turkish MaCriCare data for this secondary analysis was authorised under a data-sharing agreement dated 11 February 2025. The secondary analysis was subsequently approved by the Gazi University Ethics Committee (approval no. 193, 24 October 2025). This study used anonymised unit-level data from the Turkish component of the main MaCriCare study and included no patient-level or personal data.

## Results

### Participating units and estimated delivery coverage

Completed and validated responses were available from 140 of 403 eligible Turkish obstetric units (34.7%). The largest annual delivery-volume categories were 501–1,500 deliveries, comprising 45 units (32.1%), and 1,501–3,000 deliveries, comprising 42 units (30.0%). For the public/private sector question, 102 units (72.9%) selected ‘Public’, 26 (18.6%) selected ‘Private’ and 12 (8.6%) selected ‘Not applicable’. Tertiary-level care was reported by 86 units (61.4%), secondary-level care by 33 (23.6%), primary-level care by 19 (13.6%) and another level of care by 2 (1.4%). University status was reported as ‘Yes’ by 72 units (51.4%), ‘No’ by 65 (46.4%) and ‘Other’ by 3 (2.1%). Overall, 70 units (50.0%) were teaching hospitals. Based on their reported annual delivery-volume categories, the participating units represented approximately 305,250 deliveries per year, equivalent to 27.4% of the 1,112,859 live births recorded nationally in 2020 [4].

### Same-hospital ICU access and early-warning systems

Same-hospital ICU access was reported by 110/140 Turkish units (78.6%, 95% CI 70.8–85.1%) and 818/993 non-Türkiye units (82.4%). Among Turkish units, access was reported by 76/102 public units (74.5%) and 23/26 private units (88.5%); 21/33 secondary-level units (63.6%) and 76/86 tertiary-level units (88.4%); and 64/72 university hospitals (88.9%) and 43/65 non-university hospitals (66.2%). The complete ‘Yes’, ‘No’ and ‘Other’ distributions by unit characteristic and annual delivery volume are presented in Table 1.

**Table 1.** Same-hospital intensive-care unit access according to unit characteristics and annual delivery volume.

Characteristic	Türkiye: Yes	Türkiye: No	Türkiye: Other	Non-Türkiye: Yes	Non-Türkiye: No	Non-Türkiye: Other
Overall	110/140 (78.6%)	26/140 (18.6%)	4/140 (2.9%)	818/993 (82.4%)	149/993 (15.0%)	26/993 (2.6%)
Annual delivery volume						
1–500	13/25 (52.0%)	9/25 (36.0%)	3/25 (12.0%)	79/131 (60.3%)	41/131 (31.3%)	11/131 (8.4%)
501–1,500	39/45 (86.7%)	6/45 (13.3%)	0/45 (0.0%)	236/298 (79.2%)	54/298 (18.1%)	8/298 (2.7%)
1,501–3,000	35/42 (83.3%)	7/42 (16.7%)	0/42 (0.0%)	284/319 (89.0%)	31/319 (9.7%)	4/319 (1.3%)
3,001–4,500	9/11 (81.8%)	1/11 (9.1%)	1/11 (9.1%)	125/135 (92.6%)	9/135 (6.7%)	1/135 (0.7%)
4,501–6,000	5/7	2/7	0/7	54/59 (91.5%)	5/59 (8.5%)	0/59 (0.0%)
6,001–7,500	3/3	0/3	0/3	19/25 (76.0%)	4/25 (16.0%)	2/25 (8.0%)
>7,500	6/7	1/7	0/7	21/26 (80.8%)	5/26 (19.2%)	0/26 (0.0%)
Public/private sector						
Public	76/102 (74.5%)	22/102 (21.6%)	4/102 (3.9%)	744/885 (84.1%)	116/885 (13.1%)	25/885 (2.8%)
Private	23/26 (88.5%)	3/26 (11.5%)	0/26 (0.0%)	59/90 (65.6%)	30/90 (33.3%)	1/90 (1.1%)
Not applicable	11/12 (91.7%)	1/12 (8.3%)	0/12 (0.0%)	15/18 (83.3%)	3/18 (16.7%)	0/18 (0.0%)
Highest level of care provided						
Primary	12/19 (63.2%)	6/19 (31.6%)	1/19 (5.3%)	165/235 (70.2%)	59/235 (25.1%)	11/235 (4.7%)
Secondary	21/33 (63.6%)	10/33 (30.3%)	2/33 (6.1%)	307/374 (82.1%)	58/374 (15.5%)	9/374 (2.4%)
Tertiary	76/86 (88.4%)	9/86 (10.5%)	1/86 (1.2%)	329/359 (91.6%)	24/359 (6.7%)	6/359 (1.7%)
Other	1/2	1/2	0/2	17/25 (68.0%)	8/25 (32.0%)	0/25 (0.0%)
University hospital						
Yes	64/72 (88.9%)	8/72 (11.1%)	0/72 (0.0%)	276/309 (89.3%)	27/309 (8.7%)	6/309 (1.9%)
No	43/65 (66.2%)	18/65 (27.7%)	4/65 (6.2%)	535/675 (79.3%)	121/675 (17.9%)	19/675 (2.8%)
Other	3/3	0/3	0/3	7/9 (77.8%)	1/9 (11.1%)	1/9 (11.1%)
Teaching hospital						
Yes	63/70 (90.0%)	7/70 (10.0%)	0/70 (0.0%)	630/727 (86.7%)	85/727 (11.7%)	12/727 (1.7%)
No	47/70 (67.1%)	19/70 (27.1%)	4/70 (5.7%)	184/258 (71.3%)	60/258 (23.3%)	14/258 (5.4%)
Other	—	—	—	4/8 (50.0%)	4/8 (50.0%)	0/8 (0.0%)

Data are *n*/*N* (%). For Turkish strata containing fewer than 10 units, percentages are omitted. The non-Türkiye comparator was calculated by subtracting the 140 Turkish units from the corresponding totals for the full 1,133-unit MaCriCare cohort. ‘Other’ responses were retained as a separate category. Percentages may not total 100% because of rounding.

Use of MEOWS or another early-warning system was reported by 39/140 Turkish units (27.9%, 95% CI 20.6–36.1%) and 352/993 non-Türkiye units (35.4%). Among the Turkish units, 94/140 (67.1%) reported that no such system was used and 7/140 (5.0%) selected ‘Other’.

### Reported care location in clinical scenarios

For pre-eclampsia, 31/140 Turkish units (22.1%) reported management within the obstetric unit before and after delivery. Care within the obstetric unit before delivery followed by separate critical care after delivery was reported by 58/140 units (41.4%), while 35/140 (25.0%) reported separate critical care before and after delivery. Transfer to another hospital was reported by 13/140 units (9.3%), and 3/140 (2.1%) selected ‘Other’.

For stable major PPH, separate critical care was reported by 87/140 Turkish units (62.1%), compared with 441/993 non-Türkiye units (44.4%). The corresponding values were 116/140 (82.9%) and 726/993 (73.1%) for unstable major PPH, and 121/140 (86.4%) and 716/993 (72.1%) for sepsis. Transfer to another hospital was reported by 6/140 Turkish units (4.3%) for stable major PPH, 20/140 (14.3%) for unstable major PPH and 16/140 (11.4%) for sepsis. The corresponding non-Türkiye values were 43/993 (4.3%), 130/993 (13.1%) and 140/993 (14.1%), respectively. Complete distributions for all four scenarios are presented in Table 2.

**Table 2.** Reported care locations for four clinical scenarios among Turkish and non-Türkiye MaCriCare units.

Clinical scenario and reported care location	Türkiye, *n*/*N* (%)	Non-Türkiye, *n*/*N* (%)
Pre-eclampsia		
Care in the obstetric unit before and after delivery	31/140 (22.1%)	333/993 (33.5%)
Care in the obstetric unit before delivery followed by separate critical care after delivery	58/140 (41.4%)	314/993 (31.6%)
Separate critical care before and after delivery	35/140 (25.0%)	195/993 (19.6%)
Transfer to another hospital	13/140 (9.3%)	132/993 (13.3%)
Other	3/140 (2.1%)	19/993 (1.9%)
Stable major PPH		
Care entirely within the obstetric unit	45/140 (32.1%)	483/993 (48.6%)
Separate critical care	87/140 (62.1%)	441/993 (44.4%)
Transfer to another hospital	6/140 (4.3%)	43/993 (4.3%)
Other	2/140 (1.4%)	26/993 (2.6%)
Unstable major PPH		
Care entirely within the obstetric unit	2/140 (1.4%)	134/993 (13.5%)
Separate critical care	116/140 (82.9%)	726/993 (73.1%)
Transfer to another hospital	20/140 (14.3%)	130/993 (13.1%)
Other	2/140 (1.4%)	3/993 (0.3%)
Sepsis		
Care entirely within the obstetric unit	1/140 (0.7%)	127/993 (12.8%)
Separate critical care	121/140 (86.4%)	716/993 (72.1%)
Transfer to another hospital	16/140 (11.4%)	140/993 (14.1%)
Other	2/140 (1.4%)	10/993 (1.0%)

Data are *n*/*N* (%). Responses were mutually exclusive within each scenario and describe the unit’s usual reported care location. The non-Türkiye comparator was calculated by subtracting the 140 Turkish units from the corresponding totals for the full 1,133-unit MaCriCare cohort. ‘Other’ responses were retained as a separate category. Percentages may not total 100% because of rounding. PPH, postpartum haemorrhage.

### Clinical services and resources

Among the 31 clinical services and resources assessed, cardiac output monitoring was reported as unavailable by 42/140 Turkish units (30.0%), cell salvage by 66/140 (47.1%) and viscoelastic testing by 94/140 (67.1%). The corresponding values among non-Türkiye units were 153/993 (15.4%), 311/993 (31.3%) and 386/993 (38.9%), respectively. Invasive blood-pressure monitoring was reported in the same ward or an adjoining area or in the same building by 137/140 Turkish units (97.9%), and laboratory services by 128/140 (91.4%). The complete location distributions are presented in Table 3, with results by annual delivery volume provided in Supplementary Table S1.

**Table 3.** Location of available equipment, facilities, and specialized clinical services in Türkiye and non-Türkiye MaCriCare obstetric units.

Clinical service/resource	Cohort	Same ward/adjoining	Same building	Different building	Different hospital	Not available
Advanced monitoring						
Invasive blood pressure monitoring	Türkiye (*N* = 140)	78/140 (55.7%)	59/140 (42.1%)	1/140 (0.7%)	0/140 (0.0%)	2/140 (1.4%)
	Non-Türkiye (*N* = 993)	524/993 (52.8%)	256/993 (25.8%)	40/993 (4.0%)	30/993 (3.0%)	143/993 (14.4%)
Cardiac output monitoring	Türkiye (*N* = 140)	27/140 (19.3%)	58/140 (41.4%)	10/140 (7.1%)	3/140 (2.1%)	42/140 (30.0%)
	Non-Türkiye (*N* = 993)	260/993 (26.2%)	383/993 (38.6%)	119/993 (12.0%)	78/993 (7.9%)	153/993 (15.4%)
Advanced cardiorespiratory and renal therapies						
Intra-aortic balloon pump	Türkiye (*N* = 140)	15/140 (10.7%)	61/140 (43.6%)	22/140 (15.7%)	7/140 (5.0%)	35/140 (25.0%)
	Non-Türkiye (*N* = 993)	45/993 (4.5%)	198/993 (19.9%)	130/993 (13.1%)	232/993 (23.4%)	388/993 (39.1%)
Invasive ventilation	Türkiye (*N* = 140)	34/140 (24.3%)	93/140 (66.4%)	8/140 (5.7%)	2/140 (1.4%)	3/140 (2.1%)
	Non-Türkiye (*N* = 993)	462/993 (46.5%)	406/993 (40.9%)	92/993 (9.3%)	25/993 (2.5%)	8/993 (0.8%)
Non-invasive ventilation	Türkiye (*N* = 140)	46/140 (32.9%)	84/140 (60.0%)	7/140 (5.0%)	2/140 (1.4%)	1/140 (0.7%)
	Non-Türkiye (*N* = 993)	461/993 (46.4%)	409/993 (41.2%)	84/993 (8.5%)	20/993 (2.0%)	19/993 (1.9%)
ECMO	Türkiye (*N* = 140)	5/140 (3.6%)	46/140 (32.9%)	17/140 (12.1%)	11/140 (7.9%)	61/140 (43.6%)
	Non-Türkiye (*N* = 993)	21/993 (2.1%)	123/993 (12.4%)	103/993 (10.4%)	373/993 (37.6%)	373/993 (37.6%)
Renal replacement therapy	Türkiye (*N* = 140)	15/140 (10.7%)	90/140 (64.3%)	18/140 (12.9%)	3/140 (2.1%)	14/140 (10.0%)
	Non-Türkiye (*N* = 993)	73/993 (7.4%)	420/993 (42.3%)	211/993 (21.2%)	155/993 (15.6%)	134/993 (13.5%)
Advanced pharmacological therapies						
Hypotensive agent infusions	Türkiye (*N* = 140)	91/140 (65.0%)	47/140 (33.6%)	2/140 (1.4%)	0/140 (0.0%)	0/140 (0.0%)
	Non-Türkiye (*N* = 993)	772/993 (77.7%)	171/993 (17.2%)	11/993 (1.1%)	8/993 (0.8%)	31/993 (3.1%)
Vasopressor infusions	Türkiye (*N* = 140)	83/140 (59.3%)	55/140 (39.3%)	2/140 (1.4%)	0/140 (0.0%)	0/140 (0.0%)
	Non-Türkiye (*N* = 993)	714/993 (71.9%)	236/993 (23.8%)	26/993 (2.6%)	9/993 (0.9%)	8/993 (0.8%)
Access to blood products and cell saver						
Transfusion services (blood bank)	Türkiye (*N* = 140)	18/140 (12.9%)	101/140 (72.1%)	13/140 (9.3%)	3/140 (2.1%)	5/140 (3.6%)
	Non-Türkiye (*N* = 993)	169/993 (17.0%)	479/993 (48.2%)	229/993 (23.1%)	92/993 (9.3%)	24/993 (2.4%)
Cell saver	Türkiye (*N* = 140)	20/140 (14.3%)	43/140 (30.7%)	10/140 (7.1%)	1/140 (0.7%)	66/140 (47.1%)
	Non-Türkiye (*N* = 993)	293/993 (29.5%)	262/993 (26.4%)	57/993 (5.7%)	70/993 (7.0%)	311/993 (31.3%)
Point-of care testing						
POC for haemoglobin	Türkiye (*N* = 140)	18/140 (12.9%)	82/140 (58.6%)	6/140 (4.3%)	1/140 (0.7%)	33/140 (23.6%)
	Non-Türkiye (*N* = 993)	494/993 (49.7%)	312/993 (31.4%)	65/993 (6.5%)	8/993 (0.8%)	114/993 (11.5%)
POC blood gas analysis	Türkiye (*N* = 140)	26/140 (18.6%)	97/140 (69.3%)	6/140 (4.3%)	0/140 (0.0%)	11/140 (7.9%)
	Non-Türkiye (*N* = 993)	441/993 (44.4%)	370/993 (37.3%)	60/993 (6.0%)	16/993 (1.6%)	106/993 (10.7%)
POC lactate	Türkiye (*N* = 140)	25/140 (17.9%)	84/140 (60.0%)	5/140 (3.6%)	1/140 (0.7%)	25/140 (17.9%)
	Non-Türkiye (*N* = 993)	452/993 (45.5%)	327/993 (32.9%)	68/993 (6.8%)	17/993 (1.7%)	129/993 (13.0%)
POC glucose	Türkiye (*N* = 140)	86/140 (61.4%)	50/140 (35.7%)	0/140 (0.0%)	1/140 (0.7%)	3/140 (2.1%)
	Non-Türkiye (*N* = 993)	653/993 (65.8%)	241/993 (24.3%)	49/993 (4.9%)	7/993 (0.7%)	43/993 (4.3%)
POC INR	Türkiye (*N* = 140)	16/140 (11.4%)	98/140 (70.0%)	6/140 (4.3%)	1/140 (0.7%)	19/140 (13.6%)
	Non-Türkiye (*N* = 993)	142/993 (14.3%)	441/993 (44.4%)	155/993 (15.6%)	23/993 (2.3%)	232/993 (23.4%)
Thromboelastometry (TEG/ROTEM)	Türkiye (*N* = 140)	2/140 (1.4%)	32/140 (22.9%)	7/140 (5.0%)	5/140 (3.6%)	94/140 (67.1%)
	Non-Türkiye (*N* = 993)	156/993 (15.7%)	214/993 (21.6%)	101/993 (10.2%)	136/993 (13.7%)	386/993 (38.9%)
Diagnostic imaging						
Computed tomography	Türkiye (*N* = 140)	8/140 (5.7%)	111/140 (79.3%)	15/140 (10.7%)	2/140 (1.4%)	4/140 (2.9%)
	Non-Türkiye (*N* = 993)	67/993 (6.7%)	527/993 (53.1%)	235/993 (23.7%)	111/993 (11.2%)	53/993 (5.3%)
Magnetic resonance imaging	Türkiye (*N* = 140)	8/140 (5.7%)	103/140 (73.6%)	19/140 (13.6%)	3/140 (2.1%)	7/140 (5.0%)
	Non-Türkiye (*N* = 993)	54/993 (5.4%)	431/993 (43.4%)	228/993 (23.0%)	161/993 (16.2%)	119/993 (12.0%)
Ultrasound imaging equipment	Türkiye (*N* = 140)	97/140 (69.3%)	42/140 (30.0%)	1/140 (0.7%)	0/140 (0.0%)	0/140 (0.0%)
	Non-Türkiye (*N* = 993)	728/993 (73.3%)	247/993 (24.9%)	14/993 (1.4%)	0/993 (0.0%)	4/993 (0.4%)
Facilities						
Dedicated operating room for obstetric patients	Türkiye (*N* = 140)	76/140 (54.3%)	59/140 (42.1%)	2/140 (1.4%)	1/140 (0.7%)	2/140 (1.4%)
	Non-Türkiye (*N* = 993)	631/993 (63.5%)	306/993 (30.8%)	17/993 (1.7%)	5/993 (0.5%)	34/993 (3.4%)
Neonatal intensive care	Türkiye (*N* = 140)	37/140 (26.4%)	85/140 (60.7%)	5/140 (3.6%)	2/140 (1.4%)	11/140 (7.9%)
	Non-Türkiye (*N* = 993)	301/993 (30.3%)	397/993 (40.0%)	25/993 (2.5%)	180/993 (18.1%)	90/993 (9.1%)
Ability to accommodate COVID-19 suspected or confirmed patients	Türkiye (*N* = 140)	16/140 (11.4%)	91/140 (65.0%)	20/140 (14.3%)	4/140 (2.9%)	9/140 (6.4%)
	Non-Türkiye (*N* = 993)	380/993 (38.3%)	364/993 (36.7%)	79/993 (8.0%)	113/993 (11.4%)	57/993 (5.7%)
Other specialized clinical and diagnostic services						
Specialist acute stroke service	Türkiye (*N* = 140)	4/140 (2.9%)	63/140 (45.0%)	23/140 (16.4%)	5/140 (3.6%)	45/140 (32.1%)
	Non-Türkiye (*N* = 993)	33/993 (3.3%)	300/993 (30.2%)	233/993 (23.5%)	309/993 (31.1%)	118/993 (11.9%)
Specialist burns service	Türkiye (*N* = 140)	4/140 (2.9%)	29/140 (20.7%)	16/140 (11.4%)	13/140 (9.3%)	78/140 (55.7%)
	Non-Türkiye (*N* = 993)	4/993 (0.4%)	53/993 (5.3%)	90/993 (9.1%)	510/993 (51.4%)	336/993 (33.8%)
Specialist cardiothoracic service	Türkiye (*N* = 140)	6/140 (4.3%)	79/140 (56.4%)	19/140 (13.6%)	6/140 (4.3%)	30/140 (21.4%)
	Non-Türkiye (*N* = 993)	13/993 (1.3%)	116/993 (11.7%)	122/993 (12.3%)	495/993 (49.8%)	247/993 (24.9%)
Specialist neurosurgical service	Türkiye (*N* = 140)	6/140 (4.3%)	82/140 (58.6%)	25/140 (17.9%)	2/140 (1.4%)	25/140 (17.9%)
	Non-Türkiye (*N* = 993)	20/993 (2.0%)	153/993 (15.4%)	150/993 (15.1%)	459/993 (46.2%)	211/993 (21.2%)
Specialist psychiatric service	Türkiye (*N* = 140)	5/140 (3.6%)	58/140 (41.4%)	28/140 (20.0%)	9/140 (6.4%)	40/140 (28.6%)
	Non-Türkiye (*N* = 993)	34/993 (3.4%)	224/993 (22.6%)	274/993 (27.6%)	363/993 (36.6%)	98/993 (9.9%)
Specialist trauma service	Türkiye (*N* = 140)	7/140 (5.0%)	57/140 (40.7%)	18/140 (12.9%)	4/140 (2.9%)	54/140 (38.6%)
	Non-Türkiye (*N* = 993)	46/993 (4.6%)	329/993 (33.1%)	199/993 (20.0%)	301/993 (30.3%)	118/993 (11.9%)
Laboratory services	Türkiye (*N* = 140)	19/140 (13.6%)	109/140 (77.9%)	11/140 (7.9%)	0/140 (0.0%)	1/140 (0.7%)
	Non-Türkiye (*N* = 993)	181/993 (18.2%)	562/993 (56.6%)	235/993 (23.7%)	14/993 (1.4%)	1/993 (0.1%)
Interventional radiology service (24/7/365)	Türkiye (*N* = 140)	8/140 (5.7%)	72/140 (51.4%)	10/140 (7.1%)	5/140 (3.6%)	45/140 (32.1%)
	Non-Türkiye (*N* = 993)	63/993 (6.3%)	322/993 (32.4%)	172/993 (17.3%)	265/993 (26.7%)	171/993 (17.2%)

Data are *n/N* (%). For each service/resource, respondents selected one of five locations, indicating the closest routine location available to the obstetric patient. A therapy that could only be initiated locally but required transfer elsewhere for continuation was not considered routinely available at the initiating location; post-anaesthesia recovery areas were counted only when the specified therapy could be sustained for >24 h. The non-Türkiye comparator was calculated by subtracting the 140 Turkish units from the corresponding totals for the full 1,133-unit MaCriCare cohort. Percentages may not total 100% because of rounding. ECMO, extracorporeal membrane oxygenation; POC, point-of-care; INR, international normalised ratio; TEG, thromboelastography; ROTEM, rotational thromboelastometry.

### Maternal experience, follow-up and staff support

Among the 110 Turkish units with same-hospital ICU access, breastfeeding support or initiation was the most frequently reported provision, reported by 102/110 units (92.7%), compared with 661/818 non-Türkiye units (80.8%). Maternal psychological screening or support was the least frequently reported provision in Türkiye, reported by 27/110 units (24.5%), compared with 257/818 non-Türkiye units (31.4%). Results for all seven maternal and staff support provisions are presented in Table 4, with results by annual delivery volume provided in Supplementary Table S2.

**Table 4.** Support and follow-up for critical care unit patients and staff among units with same-hospital ICU access.

Provision	Türkiye, *n*/*N* (%)	Non-Türkiye, *n*/*N* (%)
Breast feeding support/initiation	102/110 (92.7%)	661/818 (80.8%)
Physical contact between mother and baby is routinely facilitated while mother is in ICU	74/110 (67.3%)	528/818 (64.5%)
Virtual contact between mother and baby is routinely facilitated while mother is in ICU (e.g. using video-calls)	61/110 (55.5%)	333/818 (40.7%)
Maternity patients are routinely given separate accommodation (‘side rooms’)	66/110 (60.0%)	398/818 (48.7%)
Maternity patients are routinely followed up after discharge home in a critical care clinic or equivalent	62/110 (56.4%)	245/818 (30.0%)
Maternity patients are routinely screened for psychological trauma either before or after discharge home	27/110 (24.5%)	257/818 (31.4%)
Debriefing/ psychological support for staff is available in the event of poor maternal outcome	59/110 (53.6%)	514/818 (62.8%)

Data are units reporting that the provision was available, *n*/*N* (%). Denominators were 110 Turkish units and 818 non-Türkiye units that reported access to an ICU within the same hospital. Results by annual delivery volume are presented in Supplementary Table S2.

## Discussion

This Türkiye-specific analysis describes maternal critical-care infrastructure and reported organisational arrangements among 140 participating obstetric units. Most units reported same-hospital ICU access, although this varied across unit characteristics. MEOWS or another early-warning system was reported by fewer than one third of units. Separate critical care within the same hospital was commonly reported for the clinical scenarios, particularly unstable major PPH and sepsis. Availability varied across the 31 clinical services and resources, while maternal psychological screening and staff support were reported less frequently than breastfeeding support and mother–baby contact.

The three previous MaCriCare reports examined these aspects of maternal critical care separately. The first described preparedness for severe maternal morbidity, including early-warning systems, ICU access and reported care locations; the second examined the availability and location of clinical services and resources; and the third addressed maternal experience, follow-up and staff support [1–3]. The present analysis brings these domains together for the participating Turkish units and provides descriptive context through comparison with the remaining non-Türkiye units in the full MaCriCare cohort. The analyses used variables from the parent MaCriCare survey, but the Türkiye and non-Türkiye values presented here were newly calculated for this secondary analysis; no previously published table or figure was reproduced or adapted.

Across the clinical scenarios, Turkish units more frequently reported care in separate critical care within the same hospital than transfer to another hospital. This pattern may reflect how maternal critical care is organised within participating hospitals. Separate critical care was also reported by 62.1% of units for stable major PPH. The survey category did not specify the level of dependency, staffing model or treatments provided in these areas. This finding should therefore be interpreted as an organisational pattern and does not indicate whether transfer to separate critical care represented necessary escalation in individual cases.

When critically ill mothers receive care outside the obstetric unit, provisions supporting maternal experience and contact with the baby become an important component of service organisation. Breastfeeding support and mother–baby contact were commonly reported, whereas maternal psychological screening and staff support following a poor maternal outcome were less consistently available. These findings suggest that physical access to critical care and the support provided during and after critical illness should be considered together when assessing maternal critical-care services.

Use of MEOWS or another early-warning system was reported by 27.9% of Turkish units and 35.4% of non-Türkiye units. These findings describe reported system use rather than adoption of a particular tool. The scoping review by Nakamura et al. highlights variation in early-warning systems and outcome definitions [8]. Obstetric early-warning systems can support recognition of maternal deterioration, but their effectiveness depends on clear escalation pathways, appropriate staffing, training and regular review of their use [7]. Future service assessment should therefore consider both the system used and how it is implemented.

Cell salvage and viscoelastic testing were among the less widely available advanced resources in Turkish units. This is relevant in Türkiye, where caesarean delivery accounts for a substantial proportion of births [5], although caesarean volume alone does not establish a need for routine cell salvage in every unit. The SALVO study, a large multicentre randomised trial of routine cell salvage during caesarean delivery, did not demonstrate a statistically significant overall reduction in donor blood transfusion [9]. This finding may favour planned access for procedures in which major blood loss is anticipated rather than routine provision for all caesarean deliveries. Similarly, viscoelastic testing is most appropriately considered as part of a defined major obstetric haemorrhage protocol, alongside clinical assessment, laboratory testing and established transfusion and haemostatic pathways, rather than as a stand-alone intervention [10–12].

Strengths of this analysis include the integration of several complementary aspects of maternal critical care, the use of the original MaCriCare survey categories and the presentation of all five reported locations for clinical services and resources: the same ward or an adjoining area, the same building, a different building, a different hospital and not available. The non-Türkiye comparator excluded all Turkish units from the full MaCriCare cohort, allowing the Turkish findings to be considered alongside a non-overlapping descriptive comparison group.

The study also has limitations. Completed and validated responses were available from 34.7% of eligible Turkish units, and information about nonresponding units was unavailable. The participating units may therefore not represent all obstetric units in Türkiye. Tertiary-level, university and teaching hospitals comprised a substantial proportion of respondents, which may limit the generalisability of the findings and could result in greater reported access to some services than would be observed nationally. Province or region was not collected, so geographic variation could not be examined. Some annual delivery-volume categories contained few units, limiting their interpretation.

The findings were based on unit-level survey responses and were not independently verified through service audit. Reported availability does not establish whether a resource was immediately accessible, appropriately staffed or available 24 h a day. Care-location categories may also have been interpreted differently between units, particularly the distinction between access in a different hospital and a service that was not available. The survey described usual organisational arrangements and did not collect patient-level management or outcomes; consequently, it cannot determine the clinical appropriateness or effects of the reported arrangements. The broad early-warning-system question could include both obstetric-specific and general early-warning tools, a limitation also acknowledged in the parent MaCriCare report [1]. Consequently, the reported proportion should not be interpreted as uptake of a specific validated obstetric tool.

These findings represent a cross-sectional assessment of obstetric critical-care services conducted between September 2021 and January 2022. During this period, national hospital capacity was subject to broader healthcare system pressures, including the SARS-CoV-2 pandemic. National health statistics indicate that the total number of hospital beds in Türkiye increased from 254,497 in 2021 to 268,359 in 2024, whereas the total number of ICU beds changed only modestly, from 48,753 to 49,010 [13,14]. Because these national aggregates do not distinguish obstetric critical-care or high-dependency capacity, they cannot establish how services changed within the participating units. The findings should therefore be interpreted as a historical assessment of the participating services rather than a current audit of Turkish obstetric care.

## Conclusions

This Türkiye-specific secondary analysis describes maternal critical-care infrastructure and reported organisational arrangements among 140 participating obstetric units. Most units reported same-hospital ICU access, while separate critical care within the same hospital was commonly reported for unstable major PPH and sepsis. Fewer than one third of units reported using MEOWS or another early-warning system, and availability varied for advanced resources, including cardiac output monitoring, cell salvage and viscoelastic testing. Breastfeeding support and mother–baby contact were commonly reported, whereas maternal psychological screening or support and staff support following a poor maternal outcome were less consistent. These findings provide a historical baseline for future assessment and service planning in Türkiye.

## Supplementary Material

Supplemental Material

Supplemental Material

## Data Availability

The aggregate data supporting the findings are provided in the manuscript and supplementary material. Access to anonymised respondent-level data may be considered on reasonable request to the corresponding author (gunaydin@gazi.edu.tr), subject to the MaCriCare data-governance arrangements and applicable approvals.
