# Supplementary material for: Maternal critical care infrastructure and organisational preparedness in Türkiye: a national analysis of the MaCriCare cross-sectional survey
Source: Ann Med. 2026 Oct 10;58(1):2742563. doi: 10.1080/07853890.2026.2742563 (PMC13656688; doi:10.1080/07853890.2026.2742563)
Supplement: Supplementary file 1 [file IANN_A_2742563_SM5628.docx]

**Supplementary Table S1. Location of available equipment, facilities, and specialized clinical services by annual delivery volume**

**Panel A. Türkiye**

| **Service/resource** | **Reported location** | **1–500** | **501–1,500** | **1,501–3,000** | **3,001–4,500** | **4,501–6,000** | **6,001–7,500** | **>7,500** |
| --- | --- | --- | --- | --- | --- | --- | --- | --- |
| **Advanced monitoring** | | | | | | | | |
| **Invasive blood pressure monitoring** | Same ward/adjoining | 15/25 (60.0%) | 24/45 (53.3%) | 21/42 (50.0%) | 7/11 (63.6%) | 6/7 | 1/3 | 4/7 |
|  | Same building | 8/25 (32.0%) | 21/45 (46.7%) | 21/42 (50.0%) | 4/11 (36.4%) | 1/7 | 1/3 | 3/7 |
|  | Different building | 0/25 (0.0%) | 0/45 (0.0%) | 0/42 (0.0%) | 0/11 (0.0%) | 0/7 | 1/3 | 0/7 |
|  | Different hospital | 0/25 (0.0%) | 0/45 (0.0%) | 0/42 (0.0%) | 0/11 (0.0%) | 0/7 | 0/3 | 0/7 |
|  | Not available | 2/25 (8.0%) | 0/45 (0.0%) | 0/42 (0.0%) | 0/11 (0.0%) | 0/7 | 0/3 | 0/7 |
| **Cardiac output monitoring** | Same ward/adjoining | 5/25 (20.0%) | 8/45 (17.8%) | 8/42 (19.0%) | 1/11 (9.1%) | 2/7 | 0/3 | 3/7 |
|  | Same building | 5/25 (20.0%) | 27/45 (60.0%) | 16/42 (38.1%) | 6/11 (54.5%) | 3/7 | 0/3 | 1/7 |
|  | Different building | 1/25 (4.0%) | 2/45 (4.4%) | 0/42 (0.0%) | 0/11 (0.0%) | 2/7 | 3/3 | 2/7 |
|  | Different hospital | 0/25 (0.0%) | 1/45 (2.2%) | 2/42 (4.8%) | 0/11 (0.0%) | 0/7 | 0/3 | 0/7 |
|  | Not available | 14/25 (56.0%) | 7/45 (15.6%) | 16/42 (38.1%) | 4/11 (36.4%) | 0/7 | 0/3 | 1/7 |
| **Advanced cardiorespiratory and renal therapies** | | | | | | | | |
| **Intra-aortic balloon pump** | Same ward/adjoining | 4/25 (16.0%) | 4/45 (8.9%) | 5/42 (11.9%) | 1/11 (9.1%) | 1/7 | 0/3 | 0/7 |
|  | Same building | 5/25 (20.0%) | 22/45 (48.9%) | 22/42 (52.4%) | 5/11 (45.5%) | 4/7 | 0/3 | 3/7 |
|  | Different building | 0/25 (0.0%) | 7/45 (15.6%) | 4/42 (9.5%) | 2/11 (18.2%) | 2/7 | 3/3 | 4/7 |
|  | Different hospital | 0/25 (0.0%) | 3/45 (6.7%) | 3/42 (7.1%) | 1/11 (9.1%) | 0/7 | 0/3 | 0/7 |
|  | Not available | 16/25 (64.0%) | 9/45 (20.0%) | 8/42 (19.0%) | 2/11 (18.2%) | 0/7 | 0/3 | 0/7 |
| **Invasive ventilation** | Same ward/adjoining | 11/25 (44.0%) | 9/45 (20.0%) | 9/42 (21.4%) | 2/11 (18.2%) | 1/7 | 0/3 | 2/7 |
|  | Same building | 11/25 (44.0%) | 34/45 (75.6%) | 28/42 (66.7%) | 9/11 (81.8%) | 5/7 | 2/3 | 4/7 |
|  | Different building | 0/25 (0.0%) | 2/45 (4.4%) | 3/42 (7.1%) | 0/11 (0.0%) | 1/7 | 1/3 | 1/7 |
|  | Different hospital | 0/25 (0.0%) | 0/45 (0.0%) | 2/42 (4.8%) | 0/11 (0.0%) | 0/7 | 0/3 | 0/7 |
|  | Not available | 3/25 (12.0%) | 0/45 (0.0%) | 0/42 (0.0%) | 0/11 (0.0%) | 0/7 | 0/3 | 0/7 |
| **Non-invasive ventilation** | Same ward/adjoining | 13/25 (52.0%) | 13/45 (28.9%) | 13/42 (31.0%) | 2/11 (18.2%) | 2/7 | 0/3 | 3/7 |
|  | Same building | 11/25 (44.0%) | 31/45 (68.9%) | 24/42 (57.1%) | 9/11 (81.8%) | 4/7 | 2/3 | 3/7 |
|  | Different building | 0/25 (0.0%) | 1/45 (2.2%) | 3/42 (7.1%) | 0/11 (0.0%) | 1/7 | 1/3 | 1/7 |
|  | Different hospital | 0/25 (0.0%) | 0/45 (0.0%) | 2/42 (4.8%) | 0/11 (0.0%) | 0/7 | 0/3 | 0/7 |
|  | Not available | 1/25 (4.0%) | 0/45 (0.0%) | 0/42 (0.0%) | 0/11 (0.0%) | 0/7 | 0/3 | 0/7 |
| **ECMO** | Same ward/adjoining | 1/25 (4.0%) | 2/45 (4.4%) | 2/42 (4.8%) | 0/11 (0.0%) | 0/7 | 0/3 | 0/7 |
|  | Same building | 5/25 (20.0%) | 18/45 (40.0%) | 15/42 (35.7%) | 3/11 (27.3%) | 3/7 | 0/3 | 2/7 |
|  | Different building | 2/25 (8.0%) | 7/45 (15.6%) | 0/42 (0.0%) | 2/11 (18.2%) | 1/7 | 2/3 | 3/7 |
|  | Different hospital | 0/25 (0.0%) | 5/45 (11.1%) | 3/42 (7.1%) | 1/11 (9.1%) | 1/7 | 0/3 | 1/7 |
|  | Not available | 17/25 (68.0%) | 13/45 (28.9%) | 22/42 (52.4%) | 5/11 (45.5%) | 2/7 | 1/3 | 1/7 |
| **Renal replacement therapy** | Same ward/adjoining | 6/25 (24.0%) | 4/45 (8.9%) | 3/42 (7.1%) | 1/11 (9.1%) | 1/7 | 0/3 | 0/7 |
|  | Same building | 8/25 (32.0%) | 32/45 (71.1%) | 32/42 (76.2%) | 8/11 (72.7%) | 4/7 | 0/3 | 6/7 |
|  | Different building | 2/25 (8.0%) | 6/45 (13.3%) | 3/42 (7.1%) | 1/11 (9.1%) | 2/7 | 3/3 | 1/7 |
|  | Different hospital | 0/25 (0.0%) | 1/45 (2.2%) | 2/42 (4.8%) | 0/11 (0.0%) | 0/7 | 0/3 | 0/7 |
|  | Not available | 9/25 (36.0%) | 2/45 (4.4%) | 2/42 (4.8%) | 1/11 (9.1%) | 0/7 | 0/3 | 0/7 |
| **Advanced pharmacological therapies** | | | | | | | | |
| **Hypotensive agent infusions** | Same ward/adjoining | 20/25 (80.0%) | 27/45 (60.0%) | 27/42 (64.3%) | 6/11 (54.5%) | 5/7 | 1/3 | 5/7 |
|  | Same building | 5/25 (20.0%) | 18/45 (40.0%) | 14/42 (33.3%) | 5/11 (45.5%) | 2/7 | 1/3 | 2/7 |
|  | Different building | 0/25 (0.0%) | 0/45 (0.0%) | 1/42 (2.4%) | 0/11 (0.0%) | 0/7 | 1/3 | 0/7 |
|  | Different hospital | 0/25 (0.0%) | 0/45 (0.0%) | 0/42 (0.0%) | 0/11 (0.0%) | 0/7 | 0/3 | 0/7 |
|  | Not available | 0/25 (0.0%) | 0/45 (0.0%) | 0/42 (0.0%) | 0/11 (0.0%) | 0/7 | 0/3 | 0/7 |
| **Vasopressor infusions** | Same ward/adjoining | 19/25 (76.0%) | 24/45 (53.3%) | 24/42 (57.1%) | 6/11 (54.5%) | 4/7 | 1/3 | 5/7 |
|  | Same building | 6/25 (24.0%) | 21/45 (46.7%) | 17/42 (40.5%) | 5/11 (45.5%) | 3/7 | 1/3 | 2/7 |
|  | Different building | 0/25 (0.0%) | 0/45 (0.0%) | 1/42 (2.4%) | 0/11 (0.0%) | 0/7 | 1/3 | 0/7 |
|  | Different hospital | 0/25 (0.0%) | 0/45 (0.0%) | 0/42 (0.0%) | 0/11 (0.0%) | 0/7 | 0/3 | 0/7 |
|  | Not available | 0/25 (0.0%) | 0/45 (0.0%) | 0/42 (0.0%) | 0/11 (0.0%) | 0/7 | 0/3 | 0/7 |
| **Access to blood products and cell saver** | | | | | | | | |
| **Transfusion services (blood bank)** | Same ward/adjoining | 4/25 (16.0%) | 8/45 (17.8%) | 3/42 (7.1%) | 1/11 (9.1%) | 1/7 | 0/3 | 1/7 |
|  | Same building | 15/25 (60.0%) | 32/45 (71.1%) | 33/42 (78.6%) | 8/11 (72.7%) | 5/7 | 3/3 | 5/7 |
|  | Different building | 1/25 (4.0%) | 2/45 (4.4%) | 6/42 (14.3%) | 2/11 (18.2%) | 1/7 | 0/3 | 1/7 |
|  | Different hospital | 2/25 (8.0%) | 1/45 (2.2%) | 0/42 (0.0%) | 0/11 (0.0%) | 0/7 | 0/3 | 0/7 |
|  | Not available | 3/25 (12.0%) | 2/45 (4.4%) | 0/42 (0.0%) | 0/11 (0.0%) | 0/7 | 0/3 | 0/7 |
| **Cell saver** | Same ward/adjoining | 7/25 (28.0%) | 5/45 (11.1%) | 4/42 (9.5%) | 0/11 (0.0%) | 3/7 | 0/3 | 1/7 |
|  | Same building | 6/25 (24.0%) | 15/45 (33.3%) | 14/42 (33.3%) | 4/11 (36.4%) | 2/7 | 1/3 | 1/7 |
|  | Different building | 0/25 (0.0%) | 2/45 (4.4%) | 4/42 (9.5%) | 2/11 (18.2%) | 1/7 | 0/3 | 1/7 |
|  | Different hospital | 0/25 (0.0%) | 0/45 (0.0%) | 1/42 (2.4%) | 0/11 (0.0%) | 0/7 | 0/3 | 0/7 |
|  | Not available | 12/25 (48.0%) | 23/45 (51.1%) | 19/42 (45.2%) | 5/11 (45.5%) | 1/7 | 2/3 | 4/7 |
| **Point-of care testing** | | | | | | | | |
| **POC for Haemoglobin** | Same ward/adjoining | 3/25 (12.0%) | 5/45 (11.1%) | 6/42 (14.3%) | 1/11 (9.1%) | 3/7 | 0/3 | 0/7 |
|  | Same building | 14/25 (56.0%) | 28/45 (62.2%) | 25/42 (59.5%) | 6/11 (54.5%) | 4/7 | 1/3 | 4/7 |
|  | Different building | 0/25 (0.0%) | 1/45 (2.2%) | 4/42 (9.5%) | 0/11 (0.0%) | 0/7 | 0/3 | 1/7 |
|  | Different hospital | 0/25 (0.0%) | 1/45 (2.2%) | 0/42 (0.0%) | 0/11 (0.0%) | 0/7 | 0/3 | 0/7 |
|  | Not available | 8/25 (32.0%) | 10/45 (22.2%) | 7/42 (16.7%) | 4/11 (36.4%) | 0/7 | 2/3 | 2/7 |
| **POC Blood gas analysis** | Same ward/adjoining | 4/25 (16.0%) | 8/45 (17.8%) | 8/42 (19.0%) | 3/11 (27.3%) | 2/7 | 0/3 | 1/7 |
|  | Same building | 16/25 (64.0%) | 33/45 (73.3%) | 28/42 (66.7%) | 7/11 (63.6%) | 4/7 | 3/3 | 6/7 |
|  | Different building | 1/25 (4.0%) | 2/45 (4.4%) | 2/42 (4.8%) | 0/11 (0.0%) | 1/7 | 0/3 | 0/7 |
|  | Different hospital | 0/25 (0.0%) | 0/45 (0.0%) | 0/42 (0.0%) | 0/11 (0.0%) | 0/7 | 0/3 | 0/7 |
|  | Not available | 4/25 (16.0%) | 2/45 (4.4%) | 4/42 (9.5%) | 1/11 (9.1%) | 0/7 | 0/3 | 0/7 |
| **POC lactate** | Same ward/adjoining | 3/25 (12.0%) | 7/45 (15.6%) | 8/42 (19.0%) | 3/11 (27.3%) | 2/7 | 0/3 | 2/7 |
|  | Same building | 10/25 (40.0%) | 28/45 (62.2%) | 27/42 (64.3%) | 7/11 (63.6%) | 5/7 | 3/3 | 4/7 |
|  | Different building | 0/25 (0.0%) | 1/45 (2.2%) | 4/42 (9.5%) | 0/11 (0.0%) | 0/7 | 0/3 | 0/7 |
|  | Different hospital | 0/25 (0.0%) | 1/45 (2.2%) | 0/42 (0.0%) | 0/11 (0.0%) | 0/7 | 0/3 | 0/7 |
|  | Not available | 12/25 (48.0%) | 8/45 (17.8%) | 3/42 (7.1%) | 1/11 (9.1%) | 0/7 | 0/3 | 1/7 |
| **POC glucose** | Same ward/adjoining | 15/25 (60.0%) | 27/45 (60.0%) | 26/42 (61.9%) | 6/11 (54.5%) | 4/7 | 3/3 | 5/7 |
|  | Same building | 7/25 (28.0%) | 17/45 (37.8%) | 16/42 (38.1%) | 5/11 (45.5%) | 3/7 | 0/3 | 2/7 |
|  | Different building | 0/25 (0.0%) | 0/45 (0.0%) | 0/42 (0.0%) | 0/11 (0.0%) | 0/7 | 0/3 | 0/7 |
|  | Different hospital | 0/25 (0.0%) | 1/45 (2.2%) | 0/42 (0.0%) | 0/11 (0.0%) | 0/7 | 0/3 | 0/7 |
|  | Not available | 3/25 (12.0%) | 0/45 (0.0%) | 0/42 (0.0%) | 0/11 (0.0%) | 0/7 | 0/3 | 0/7 |
| **POC INR** | Same ward/adjoining | 4/25 (16.0%) | 3/45 (6.7%) | 3/42 (7.1%) | 1/11 (9.1%) | 2/7 | 0/3 | 3/7 |
|  | Same building | 14/25 (56.0%) | 35/45 (77.8%) | 32/42 (76.2%) | 7/11 (63.6%) | 5/7 | 3/3 | 2/7 |
|  | Different building | 0/25 (0.0%) | 1/45 (2.2%) | 4/42 (9.5%) | 1/11 (9.1%) | 0/7 | 0/3 | 0/7 |
|  | Different hospital | 0/25 (0.0%) | 1/45 (2.2%) | 0/42 (0.0%) | 0/11 (0.0%) | 0/7 | 0/3 | 0/7 |
|  | Not available | 7/25 (28.0%) | 5/45 (11.1%) | 3/42 (7.1%) | 2/11 (18.2%) | 0/7 | 0/3 | 2/7 |
| **Thromboelastometry (TEG/ROTEM)** | Same ward/adjoining | 0/25 (0.0%) | 0/45 (0.0%) | 2/42 (4.8%) | 0/11 (0.0%) | 0/7 | 0/3 | 0/7 |
|  | Same building | 1/25 (4.0%) | 14/45 (31.1%) | 10/42 (23.8%) | 3/11 (27.3%) | 3/7 | 0/3 | 1/7 |
|  | Different building | 0/25 (0.0%) | 0/45 (0.0%) | 2/42 (4.8%) | 2/11 (18.2%) | 0/7 | 2/3 | 1/7 |
|  | Different hospital | 2/25 (8.0%) | 2/45 (4.4%) | 1/42 (2.4%) | 0/11 (0.0%) | 0/7 | 0/3 | 0/7 |
|  | Not available | 22/25 (88.0%) | 29/45 (64.4%) | 27/42 (64.3%) | 6/11 (54.5%) | 4/7 | 1/3 | 5/7 |
| **Diagnostic imaging** | | | | | | | | |
| **Computed Tomography** | Same ward/adjoining | 3/25 (12.0%) | 1/45 (2.2%) | 1/42 (2.4%) | 2/11 (18.2%) | 1/7 | 0/3 | 0/7 |
|  | Same building | 18/25 (72.0%) | 40/45 (88.9%) | 33/42 (78.6%) | 8/11 (72.7%) | 4/7 | 2/3 | 6/7 |
|  | Different building | 1/25 (4.0%) | 4/45 (8.9%) | 5/42 (11.9%) | 1/11 (9.1%) | 2/7 | 1/3 | 1/7 |
|  | Different hospital | 0/25 (0.0%) | 0/45 (0.0%) | 2/42 (4.8%) | 0/11 (0.0%) | 0/7 | 0/3 | 0/7 |
|  | Not available | 3/25 (12.0%) | 0/45 (0.0%) | 1/42 (2.4%) | 0/11 (0.0%) | 0/7 | 0/3 | 0/7 |
| **Magnetic Resonance Imaging** | Same ward/adjoining | 3/25 (12.0%) | 1/45 (2.2%) | 1/42 (2.4%) | 2/11 (18.2%) | 1/7 | 0/3 | 0/7 |
|  | Same building | 15/25 (60.0%) | 36/45 (80.0%) | 34/42 (81.0%) | 8/11 (72.7%) | 4/7 | 1/3 | 5/7 |
|  | Different building | 1/25 (4.0%) | 7/45 (15.6%) | 4/42 (9.5%) | 1/11 (9.1%) | 2/7 | 2/3 | 2/7 |
|  | Different hospital | 1/25 (4.0%) | 0/45 (0.0%) | 2/42 (4.8%) | 0/11 (0.0%) | 0/7 | 0/3 | 0/7 |
|  | Not available | 5/25 (20.0%) | 1/45 (2.2%) | 1/42 (2.4%) | 0/11 (0.0%) | 0/7 | 0/3 | 0/7 |
| **Ultrasound imaging equipment** | Same ward/adjoining | 15/25 (60.0%) | 32/45 (71.1%) | 30/42 (71.4%) | 7/11 (63.6%) | 6/7 | 2/3 | 5/7 |
|  | Same building | 9/25 (36.0%) | 13/45 (28.9%) | 12/42 (28.6%) | 4/11 (36.4%) | 1/7 | 1/3 | 2/7 |
|  | Different building | 1/25 (4.0%) | 0/45 (0.0%) | 0/42 (0.0%) | 0/11 (0.0%) | 0/7 | 0/3 | 0/7 |
|  | Different hospital | 0/25 (0.0%) | 0/45 (0.0%) | 0/42 (0.0%) | 0/11 (0.0%) | 0/7 | 0/3 | 0/7 |
|  | Not available | 0/25 (0.0%) | 0/45 (0.0%) | 0/42 (0.0%) | 0/11 (0.0%) | 0/7 | 0/3 | 0/7 |
| **Facilities** | | | | | | | | |
| **Dedicated operating room for obstetric patients** | Same ward/adjoining | 14/25 (56.0%) | 21/45 (46.7%) | 22/42 (52.4%) | 7/11 (63.6%) | 6/7 | 2/3 | 4/7 |
|  | Same building | 9/25 (36.0%) | 21/45 (46.7%) | 20/42 (47.6%) | 4/11 (36.4%) | 1/7 | 1/3 | 3/7 |
|  | Different building | 0/25 (0.0%) | 2/45 (4.4%) | 0/42 (0.0%) | 0/11 (0.0%) | 0/7 | 0/3 | 0/7 |
|  | Different hospital | 1/25 (4.0%) | 0/45 (0.0%) | 0/42 (0.0%) | 0/11 (0.0%) | 0/7 | 0/3 | 0/7 |
|  | Not available | 1/25 (4.0%) | 1/45 (2.2%) | 0/42 (0.0%) | 0/11 (0.0%) | 0/7 | 0/3 | 0/7 |
| **Neonatal Intensive Care** | Same ward/adjoining | 9/25 (36.0%) | 11/45 (24.4%) | 6/42 (14.3%) | 5/11 (45.5%) | 4/7 | 1/3 | 1/7 |
|  | Same building | 7/25 (28.0%) | 29/45 (64.4%) | 35/42 (83.3%) | 4/11 (36.4%) | 2/7 | 2/3 | 6/7 |
|  | Different building | 0/25 (0.0%) | 2/45 (4.4%) | 0/42 (0.0%) | 2/11 (18.2%) | 1/7 | 0/3 | 0/7 |
|  | Different hospital | 1/25 (4.0%) | 1/45 (2.2%) | 0/42 (0.0%) | 0/11 (0.0%) | 0/7 | 0/3 | 0/7 |
|  | Not available | 8/25 (32.0%) | 2/45 (4.4%) | 1/42 (2.4%) | 0/11 (0.0%) | 0/7 | 0/3 | 0/7 |
| **Ability to accommodate COVID-19 suspected or confirmed patients** | Same ward/adjoining | 3/25 (12.0%) | 2/45 (4.4%) | 6/42 (14.3%) | 3/11 (27.3%) | 1/7 | 0/3 | 1/7 |
|  | Same building | 14/25 (56.0%) | 32/45 (71.1%) | 28/42 (66.7%) | 6/11 (54.5%) | 4/7 | 2/3 | 5/7 |
|  | Different building | 1/25 (4.0%) | 8/45 (17.8%) | 6/42 (14.3%) | 2/11 (18.2%) | 2/7 | 1/3 | 0/7 |
|  | Different hospital | 2/25 (8.0%) | 1/45 (2.2%) | 1/42 (2.4%) | 0/11 (0.0%) | 0/7 | 0/3 | 0/7 |
|  | Not available | 5/25 (20.0%) | 2/45 (4.4%) | 1/42 (2.4%) | 0/11 (0.0%) | 0/7 | 0/3 | 1/7 |
| **Other Specialized Clinical and Diagnostic Services** | | | | | | | | |
| **Specialist Acute Stroke Service** | Same ward/adjoining | 0/25 (0.0%) | 1/45 (2.2%) | 1/42 (2.4%) | 1/11 (9.1%) | 1/7 | 0/3 | 0/7 |
|  | Same building | 4/25 (16.0%) | 28/45 (62.2%) | 21/42 (50.0%) | 5/11 (45.5%) | 4/7 | 0/3 | 1/7 |
|  | Different building | 1/25 (4.0%) | 6/45 (13.3%) | 6/42 (14.3%) | 2/11 (18.2%) | 2/7 | 3/3 | 3/7 |
|  | Different hospital | 1/25 (4.0%) | 0/45 (0.0%) | 3/42 (7.1%) | 0/11 (0.0%) | 0/7 | 0/3 | 1/7 |
|  | Not available | 19/25 (76.0%) | 10/45 (22.2%) | 11/42 (26.2%) | 3/11 (27.3%) | 0/7 | 0/3 | 2/7 |
| **Specialist Burns Service** | Same ward/adjoining | 1/25 (4.0%) | 1/45 (2.2%) | 1/42 (2.4%) | 0/11 (0.0%) | 1/7 | 0/3 | 0/7 |
|  | Same building | 3/25 (12.0%) | 10/45 (22.2%) | 11/42 (26.2%) | 2/11 (18.2%) | 2/7 | 0/3 | 1/7 |
|  | Different building | 1/25 (4.0%) | 4/45 (8.9%) | 5/42 (11.9%) | 1/11 (9.1%) | 2/7 | 1/3 | 2/7 |
|  | Different hospital | 1/25 (4.0%) | 3/45 (6.7%) | 6/42 (14.3%) | 1/11 (9.1%) | 0/7 | 0/3 | 2/7 |
|  | Not available | 19/25 (76.0%) | 27/45 (60.0%) | 19/42 (45.2%) | 7/11 (63.6%) | 2/7 | 2/3 | 2/7 |
| **Specialist Cardiothoracic Service** | Same ward/adjoining | 1/25 (4.0%) | 1/45 (2.2%) | 2/42 (4.8%) | 1/11 (9.1%) | 1/7 | 0/3 | 0/7 |
|  | Same building | 10/25 (40.0%) | 28/45 (62.2%) | 29/42 (69.0%) | 5/11 (45.5%) | 4/7 | 0/3 | 3/7 |
|  | Different building | 1/25 (4.0%) | 8/45 (17.8%) | 0/42 (0.0%) | 2/11 (18.2%) | 2/7 | 3/3 | 3/7 |
|  | Different hospital | 0/25 (0.0%) | 1/45 (2.2%) | 4/42 (9.5%) | 1/11 (9.1%) | 0/7 | 0/3 | 0/7 |
|  | Not available | 13/25 (52.0%) | 7/45 (15.6%) | 7/42 (16.7%) | 2/11 (18.2%) | 0/7 | 0/3 | 1/7 |
| **Specialist Neurosurgical Service** | Same ward/adjoining | 1/25 (4.0%) | 2/45 (4.4%) | 1/42 (2.4%) | 1/11 (9.1%) | 1/7 | 0/3 | 0/7 |
|  | Same building | 11/25 (44.0%) | 33/45 (73.3%) | 26/42 (61.9%) | 6/11 (54.5%) | 3/7 | 0/3 | 3/7 |
|  | Different building | 1/25 (4.0%) | 5/45 (11.1%) | 8/42 (19.0%) | 2/11 (18.2%) | 3/7 | 3/3 | 3/7 |
|  | Different hospital | 0/25 (0.0%) | 0/45 (0.0%) | 2/42 (4.8%) | 0/11 (0.0%) | 0/7 | 0/3 | 0/7 |
|  | Not available | 12/25 (48.0%) | 5/45 (11.1%) | 5/42 (11.9%) | 2/11 (18.2%) | 0/7 | 0/3 | 1/7 |
| **Specialist Psychiatric Service** | Same ward/adjoining | 1/25 (4.0%) | 1/45 (2.2%) | 1/42 (2.4%) | 2/11 (18.2%) | 0/7 | 0/3 | 0/7 |
|  | Same building | 4/25 (16.0%) | 24/45 (53.3%) | 20/42 (47.6%) | 4/11 (36.4%) | 4/7 | 0/3 | 2/7 |
|  | Different building | 3/25 (12.0%) | 5/45 (11.1%) | 9/42 (21.4%) | 3/11 (27.3%) | 3/7 | 3/3 | 2/7 |
|  | Different hospital | 1/25 (4.0%) | 3/45 (6.7%) | 4/42 (9.5%) | 0/11 (0.0%) | 0/7 | 0/3 | 1/7 |
|  | Not available | 16/25 (64.0%) | 12/45 (26.7%) | 8/42 (19.0%) | 2/11 (18.2%) | 0/7 | 0/3 | 2/7 |
| **Specialist Trauma Service** | Same ward/adjoining | 3/25 (12.0%) | 2/45 (4.4%) | 1/42 (2.4%) | 0/11 (0.0%) | 1/7 | 0/3 | 0/7 |
|  | Same building | 5/25 (20.0%) | 21/45 (46.7%) | 19/42 (45.2%) | 6/11 (54.5%) | 2/7 | 0/3 | 4/7 |
|  | Different building | 1/25 (4.0%) | 4/45 (8.9%) | 5/42 (11.9%) | 1/11 (9.1%) | 2/7 | 3/3 | 2/7 |
|  | Different hospital | 1/25 (4.0%) | 1/45 (2.2%) | 2/42 (4.8%) | 0/11 (0.0%) | 0/7 | 0/3 | 0/7 |
|  | Not available | 15/25 (60.0%) | 17/45 (37.8%) | 15/42 (35.7%) | 4/11 (36.4%) | 2/7 | 0/3 | 1/7 |
| **Laboratory Services** | Same ward/adjoining | 5/25 (20.0%) | 6/45 (13.3%) | 3/42 (7.1%) | 2/11 (18.2%) | 1/7 | 0/3 | 2/7 |
|  | Same building | 17/25 (68.0%) | 36/45 (80.0%) | 35/42 (83.3%) | 8/11 (72.7%) | 6/7 | 2/3 | 5/7 |
|  | Different building | 2/25 (8.0%) | 3/45 (6.7%) | 4/42 (9.5%) | 1/11 (9.1%) | 0/7 | 1/3 | 0/7 |
|  | Different hospital | 0/25 (0.0%) | 0/45 (0.0%) | 0/42 (0.0%) | 0/11 (0.0%) | 0/7 | 0/3 | 0/7 |
|  | Not available | 1/25 (4.0%) | 0/45 (0.0%) | 0/42 (0.0%) | 0/11 (0.0%) | 0/7 | 0/3 | 0/7 |
| **Interventional Radiology Service (24/7/365)** | Same ward/adjoining | 1/25 (4.0%) | 3/45 (6.7%) | 2/42 (4.8%) | 1/11 (9.1%) | 1/7 | 0/3 | 0/7 |
|  | Same building | 9/25 (36.0%) | 28/45 (62.2%) | 22/42 (52.4%) | 5/11 (45.5%) | 2/7 | 1/3 | 5/7 |
|  | Different building | 1/25 (4.0%) | 3/45 (6.7%) | 3/42 (7.1%) | 1/11 (9.1%) | 1/7 | 0/3 | 1/7 |
|  | Different hospital | 0/25 (0.0%) | 1/45 (2.2%) | 3/42 (7.1%) | 0/11 (0.0%) | 0/7 | 1/3 | 0/7 |
|  | Not available | 14/25 (56.0%) | 10/45 (22.2%) | 12/42 (28.6%) | 4/11 (36.4%) | 3/7 | 1/3 | 1/7 |

*Data are n/N (%), except Turkish strata containing fewer than 10 units, which are shown as n/N. Annual-delivery-volume denominators for Türkiye were 25, 45, 42, 11, 7, 3 and 7 units, respectively. For each service/resource, respondents selected one of five locations indicating the closest routine location available to the obstetric patient: same ward/adjoining, same building, different building, different hospital, or not available.*

**Panel B. Non-Türkiye**

| **Service/resource** | **Reported location** | **1–500** | **501–1,500** | **1,501–3,000** | **3,001–4,500** | **4,501–6,000** | **6,001–7,500** | **>7,500** |
| --- | --- | --- | --- | --- | --- | --- | --- | --- |
| **Advanced monitoring** | | | | | | | | |
| **Invasive blood pressure monitoring** | Same ward/adjoining | 50/131 (38.2%) | 131/298 (44.0%) | 197/319 (61.8%) | 73/135 (54.1%) | 34/59 (57.6%) | 17/25 (68.0%) | 22/26 (84.6%) |
|  | Same building | 34/131 (26.0%) | 103/298 (34.6%) | 72/319 (22.6%) | 31/135 (23.0%) | 11/59 (18.6%) | 4/25 (16.0%) | 1/26 (3.8%) |
|  | Different building | 1/131 (0.8%) | 16/298 (5.4%) | 14/319 (4.4%) | 7/135 (5.2%) | 1/59 (1.7%) | 0/25 (0.0%) | 1/26 (3.8%) |
|  | Different hospital | 4/131 (3.1%) | 9/298 (3.0%) | 7/319 (2.2%) | 5/135 (3.7%) | 4/59 (6.8%) | 0/25 (0.0%) | 1/26 (3.8%) |
|  | Not available | 42/131 (32.1%) | 39/298 (13.1%) | 29/319 (9.1%) | 19/135 (14.1%) | 9/59 (15.3%) | 4/25 (16.0%) | 1/26 (3.8%) |
| **Cardiac output monitoring** | Same ward/adjoining | 23/131 (17.6%) | 76/298 (25.5%) | 94/319 (29.5%) | 33/135 (24.4%) | 17/59 (28.8%) | 7/25 (28.0%) | 10/26 (38.5%) |
|  | Same building | 36/131 (27.5%) | 128/298 (43.0%) | 134/319 (42.0%) | 52/135 (38.5%) | 21/59 (35.6%) | 8/25 (32.0%) | 4/26 (15.4%) |
|  | Different building | 5/131 (3.8%) | 24/298 (8.1%) | 45/319 (14.1%) | 26/135 (19.3%) | 9/59 (15.3%) | 3/25 (12.0%) | 7/26 (26.9%) |
|  | Different hospital | 9/131 (6.9%) | 26/298 (8.7%) | 20/319 (6.3%) | 8/135 (5.9%) | 9/59 (15.3%) | 2/25 (8.0%) | 4/26 (15.4%) |
|  | Not available | 58/131 (44.3%) | 44/298 (14.8%) | 26/319 (8.2%) | 16/135 (11.9%) | 3/59 (5.1%) | 5/25 (20.0%) | 1/26 (3.8%) |
| **Advanced cardiorespiratory and renal therapies** | | | | | | | | |
| **Intra-aortic balloon pump** | Same ward/adjoining | 1/131 (0.8%) | 12/298 (4.0%) | 17/319 (5.3%) | 6/135 (4.4%) | 3/59 (5.1%) | 5/25 (20.0%) | 1/26 (3.8%) |
|  | Same building | 10/131 (7.6%) | 52/298 (17.4%) | 83/319 (26.0%) | 33/135 (24.4%) | 11/59 (18.6%) | 4/25 (16.0%) | 5/26 (19.2%) |
|  | Different building | 1/131 (0.8%) | 19/298 (6.4%) | 57/319 (17.9%) | 28/135 (20.7%) | 13/59 (22.0%) | 3/25 (12.0%) | 9/26 (34.6%) |
|  | Different hospital | 24/131 (18.3%) | 92/298 (30.9%) | 64/319 (20.1%) | 27/135 (20.0%) | 13/59 (22.0%) | 4/25 (16.0%) | 8/26 (30.8%) |
|  | Not available | 95/131 (72.5%) | 123/298 (41.3%) | 98/319 (30.7%) | 41/135 (30.4%) | 19/59 (32.2%) | 9/25 (36.0%) | 3/26 (11.5%) |
| **Invasive ventilation** | Same ward/adjoining | 49/131 (37.4%) | 126/298 (42.3%) | 159/319 (49.8%) | 72/135 (53.3%) | 27/59 (45.8%) | 14/25 (56.0%) | 15/26 (57.7%) |
|  | Same building | 65/131 (49.6%) | 130/298 (43.6%) | 125/319 (39.2%) | 50/135 (37.0%) | 22/59 (37.3%) | 10/25 (40.0%) | 4/26 (15.4%) |
|  | Different building | 7/131 (5.3%) | 29/298 (9.7%) | 32/319 (10.0%) | 12/135 (8.9%) | 8/59 (13.6%) | 0/25 (0.0%) | 4/26 (15.4%) |
|  | Different hospital | 5/131 (3.8%) | 10/298 (3.4%) | 3/319 (0.9%) | 1/135 (0.7%) | 2/59 (3.4%) | 1/25 (4.0%) | 3/26 (11.5%) |
|  | Not available | 5/131 (3.8%) | 3/298 (1.0%) | 0/319 (0.0%) | 0/135 (0.0%) | 0/59 (0.0%) | 0/25 (0.0%) | 0/26 (0.0%) |
| **Non-invasive ventilation** | Same ward/adjoining | 58/131 (44.3%) | 137/298 (46.0%) | 149/319 (46.7%) | 69/135 (51.1%) | 23/59 (39.0%) | 13/25 (52.0%) | 12/26 (46.2%) |
|  | Same building | 57/131 (43.5%) | 137/298 (46.0%) | 131/319 (41.1%) | 46/135 (34.1%) | 22/59 (37.3%) | 10/25 (40.0%) | 6/26 (23.1%) |
|  | Different building | 7/131 (5.3%) | 17/298 (5.7%) | 30/319 (9.4%) | 18/135 (13.3%) | 7/59 (11.9%) | 0/25 (0.0%) | 5/26 (19.2%) |
|  | Different hospital | 2/131 (1.5%) | 3/298 (1.0%) | 4/319 (1.3%) | 2/135 (1.5%) | 4/59 (6.8%) | 2/25 (8.0%) | 3/26 (11.5%) |
|  | Not available | 7/131 (5.3%) | 4/298 (1.3%) | 5/319 (1.6%) | 0/135 (0.0%) | 3/59 (5.1%) | 0/25 (0.0%) | 0/26 (0.0%) |
| **ECMO** | Same ward/adjoining | 3/131 (2.3%) | 6/298 (2.0%) | 6/319 (1.9%) | 3/135 (2.2%) | 0/59 (0.0%) | 1/25 (4.0%) | 2/26 (7.7%) |
|  | Same building | 6/131 (4.6%) | 18/298 (6.0%) | 61/319 (19.1%) | 22/135 (16.3%) | 8/59 (13.6%) | 3/25 (12.0%) | 5/26 (19.2%) |
|  | Different building | 1/131 (0.8%) | 11/298 (3.7%) | 43/319 (13.5%) | 28/135 (20.7%) | 12/59 (20.3%) | 2/25 (8.0%) | 6/26 (23.1%) |
|  | Different hospital | 33/131 (25.2%) | 145/298 (48.7%) | 101/319 (31.7%) | 52/135 (38.5%) | 22/59 (37.3%) | 11/25 (44.0%) | 9/26 (34.6%) |
|  | Not available | 88/131 (67.2%) | 118/298 (39.6%) | 108/319 (33.9%) | 30/135 (22.2%) | 17/59 (28.8%) | 8/25 (32.0%) | 4/26 (15.4%) |
| **Renal replacement therapy** | Same ward/adjoining | 5/131 (3.8%) | 23/298 (7.7%) | 27/319 (8.5%) | 6/135 (4.4%) | 4/59 (6.8%) | 4/25 (16.0%) | 4/26 (15.4%) |
|  | Same building | 39/131 (29.8%) | 143/298 (48.0%) | 155/319 (48.6%) | 56/135 (41.5%) | 14/59 (23.7%) | 8/25 (32.0%) | 5/26 (19.2%) |
|  | Different building | 6/131 (4.6%) | 51/298 (17.1%) | 71/319 (22.3%) | 44/135 (32.6%) | 26/59 (44.1%) | 2/25 (8.0%) | 11/26 (42.3%) |
|  | Different hospital | 21/131 (16.0%) | 45/298 (15.1%) | 47/319 (14.7%) | 18/135 (13.3%) | 13/59 (22.0%) | 6/25 (24.0%) | 5/26 (19.2%) |
|  | Not available | 60/131 (45.8%) | 36/298 (12.1%) | 19/319 (6.0%) | 11/135 (8.1%) | 2/59 (3.4%) | 5/25 (20.0%) | 1/26 (3.8%) |
| **Advanced pharmacological therapies** | | | | | | | | |
| **Hypotensive agent infusions** | Same ward/adjoining | 85/131 (64.9%) | 232/298 (77.9%) | 261/319 (81.8%) | 107/135 (79.3%) | 44/59 (74.6%) | 20/25 (80.0%) | 23/26 (88.5%) |
|  | Same building | 31/131 (23.7%) | 54/298 (18.1%) | 48/319 (15.0%) | 22/135 (16.3%) | 10/59 (16.9%) | 4/25 (16.0%) | 2/26 (7.7%) |
|  | Different building | 2/131 (1.5%) | 2/298 (0.7%) | 5/319 (1.6%) | 1/135 (0.7%) | 0/59 (0.0%) | 0/25 (0.0%) | 1/26 (3.8%) |
|  | Different hospital | 0/131 (0.0%) | 4/298 (1.3%) | 0/319 (0.0%) | 2/135 (1.5%) | 2/59 (3.4%) | 0/25 (0.0%) | 0/26 (0.0%) |
|  | Not available | 13/131 (9.9%) | 6/298 (2.0%) | 5/319 (1.6%) | 3/135 (2.2%) | 3/59 (5.1%) | 1/25 (4.0%) | 0/26 (0.0%) |
| **Vasopressor infusions** | Same ward/adjoining | 83/131 (63.4%) | 203/298 (68.1%) | 243/319 (76.2%) | 100/135 (74.1%) | 44/59 (74.6%) | 19/25 (76.0%) | 22/26 (84.6%) |
|  | Same building | 38/131 (29.0%) | 82/298 (27.5%) | 67/319 (21.0%) | 28/135 (20.7%) | 13/59 (22.0%) | 6/25 (24.0%) | 2/26 (7.7%) |
|  | Different building | 4/131 (3.1%) | 6/298 (2.0%) | 8/319 (2.5%) | 6/135 (4.4%) | 1/59 (1.7%) | 0/25 (0.0%) | 1/26 (3.8%) |
|  | Different hospital | 1/131 (0.8%) | 5/298 (1.7%) | 0/319 (0.0%) | 1/135 (0.7%) | 1/59 (1.7%) | 0/25 (0.0%) | 1/26 (3.8%) |
|  | Not available | 5/131 (3.8%) | 2/298 (0.7%) | 1/319 (0.3%) | 0/135 (0.0%) | 0/59 (0.0%) | 0/25 (0.0%) | 0/26 (0.0%) |
| **Access to blood products and cell saver** | | | | | | | | |
| **Transfusion services (blood bank)** | Same ward/adjoining | 15/131 (11.5%) | 46/298 (15.4%) | 62/319 (19.4%) | 27/135 (20.0%) | 10/59 (16.9%) | 3/25 (12.0%) | 6/26 (23.1%) |
|  | Same building | 74/131 (56.5%) | 163/298 (54.7%) | 140/319 (43.9%) | 54/135 (40.0%) | 24/59 (40.7%) | 14/25 (56.0%) | 10/26 (38.5%) |
|  | Different building | 13/131 (9.9%) | 58/298 (19.5%) | 83/319 (26.0%) | 41/135 (30.4%) | 21/59 (35.6%) | 4/25 (16.0%) | 9/26 (34.6%) |
|  | Different hospital | 17/131 (13.0%) | 25/298 (8.4%) | 33/319 (10.3%) | 11/135 (8.1%) | 4/59 (6.8%) | 1/25 (4.0%) | 1/26 (3.8%) |
|  | Not available | 12/131 (9.2%) | 6/298 (2.0%) | 1/319 (0.3%) | 2/135 (1.5%) | 0/59 (0.0%) | 3/25 (12.0%) | 0/26 (0.0%) |
| **Cell saver** | Same ward/adjoining | 12/131 (9.2%) | 75/298 (25.2%) | 94/319 (29.5%) | 64/135 (47.4%) | 26/59 (44.1%) | 10/25 (40.0%) | 12/26 (46.2%) |
|  | Same building | 35/131 (26.7%) | 74/298 (24.8%) | 89/319 (27.9%) | 37/135 (27.4%) | 15/59 (25.4%) | 7/25 (28.0%) | 5/26 (19.2%) |
|  | Different building | 3/131 (2.3%) | 11/298 (3.7%) | 25/319 (7.8%) | 7/135 (5.2%) | 4/59 (6.8%) | 1/25 (4.0%) | 6/26 (23.1%) |
|  | Different hospital | 8/131 (6.1%) | 32/298 (10.7%) | 23/319 (7.2%) | 3/135 (2.2%) | 2/59 (3.4%) | 1/25 (4.0%) | 1/26 (3.8%) |
|  | Not available | 73/131 (55.7%) | 106/298 (35.6%) | 88/319 (27.6%) | 24/135 (17.8%) | 12/59 (20.3%) | 6/25 (24.0%) | 2/26 (7.7%) |
| **Point-of care testing** | | | | | | | | |
| **POC for Haemoglobin** | Same ward/adjoining | 20/131 (15.3%) | 144/298 (48.3%) | 183/319 (57.4%) | 88/135 (65.2%) | 31/59 (52.5%) | 13/25 (52.0%) | 15/26 (57.7%) |
|  | Same building | 67/131 (51.1%) | 89/298 (29.9%) | 85/319 (26.6%) | 35/135 (25.9%) | 25/59 (42.4%) | 8/25 (32.0%) | 3/26 (11.5%) |
|  | Different building | 10/131 (7.6%) | 28/298 (9.4%) | 14/319 (4.4%) | 6/135 (4.4%) | 1/59 (1.7%) | 2/25 (8.0%) | 4/26 (15.4%) |
|  | Different hospital | 3/131 (2.3%) | 3/298 (1.0%) | 1/319 (0.3%) | 0/135 (0.0%) | 0/59 (0.0%) | 0/25 (0.0%) | 1/26 (3.8%) |
|  | Not available | 31/131 (23.7%) | 34/298 (11.4%) | 36/319 (11.3%) | 6/135 (4.4%) | 2/59 (3.4%) | 2/25 (8.0%) | 3/26 (11.5%) |
| **POC Blood gas analysis** | Same ward/adjoining | 25/131 (19.1%) | 117/298 (39.3%) | 169/319 (53.0%) | 73/135 (54.1%) | 29/59 (49.2%) | 11/25 (44.0%) | 17/26 (65.4%) |
|  | Same building | 65/131 (49.6%) | 111/298 (37.2%) | 112/319 (35.1%) | 44/135 (32.6%) | 25/59 (42.4%) | 10/25 (40.0%) | 3/26 (11.5%) |
|  | Different building | 4/131 (3.1%) | 30/298 (10.1%) | 13/319 (4.1%) | 7/135 (5.2%) | 1/59 (1.7%) | 2/25 (8.0%) | 3/26 (11.5%) |
|  | Different hospital | 4/131 (3.1%) | 7/298 (2.3%) | 3/319 (0.9%) | 0/135 (0.0%) | 1/59 (1.7%) | 0/25 (0.0%) | 1/26 (3.8%) |
|  | Not available | 33/131 (25.2%) | 33/298 (11.1%) | 22/319 (6.9%) | 11/135 (8.1%) | 3/59 (5.1%) | 2/25 (8.0%) | 2/26 (7.7%) |
| **POC lactate** | Same ward/adjoining | 23/131 (17.6%) | 124/298 (41.6%) | 171/319 (53.6%) | 77/135 (57.0%) | 26/59 (44.1%) | 13/25 (52.0%) | 18/26 (69.2%) |
|  | Same building | 56/131 (42.7%) | 98/298 (32.9%) | 95/319 (29.8%) | 38/135 (28.1%) | 26/59 (44.1%) | 9/25 (36.0%) | 5/26 (19.2%) |
|  | Different building | 6/131 (4.6%) | 30/298 (10.1%) | 16/319 (5.0%) | 8/135 (5.9%) | 3/59 (5.1%) | 2/25 (8.0%) | 3/26 (11.5%) |
|  | Different hospital | 5/131 (3.8%) | 6/298 (2.0%) | 4/319 (1.3%) | 1/135 (0.7%) | 1/59 (1.7%) | 0/25 (0.0%) | 0/26 (0.0%) |
|  | Not available | 41/131 (31.3%) | 40/298 (13.4%) | 33/319 (10.3%) | 11/135 (8.1%) | 3/59 (5.1%) | 1/25 (4.0%) | 0/26 (0.0%) |
| **POC glucose** | Same ward/adjoining | 50/131 (38.2%) | 190/298 (63.8%) | 235/319 (73.7%) | 102/135 (75.6%) | 41/59 (69.5%) | 16/25 (64.0%) | 19/26 (73.1%) |
|  | Same building | 56/131 (42.7%) | 66/298 (22.1%) | 64/319 (20.1%) | 28/135 (20.7%) | 16/59 (27.1%) | 7/25 (28.0%) | 4/26 (15.4%) |
|  | Different building | 7/131 (5.3%) | 25/298 (8.4%) | 8/319 (2.5%) | 3/135 (2.2%) | 1/59 (1.7%) | 2/25 (8.0%) | 3/26 (11.5%) |
|  | Different hospital | 2/131 (1.5%) | 3/298 (1.0%) | 2/319 (0.6%) | 0/135 (0.0%) | 0/59 (0.0%) | 0/25 (0.0%) | 0/26 (0.0%) |
|  | Not available | 16/131 (12.2%) | 14/298 (4.7%) | 10/319 (3.1%) | 2/135 (1.5%) | 1/59 (1.7%) | 0/25 (0.0%) | 0/26 (0.0%) |
| **POC INR** | Same ward/adjoining | 12/131 (9.2%) | 43/298 (14.4%) | 52/319 (16.3%) | 21/135 (15.6%) | 5/59 (8.5%) | 5/25 (20.0%) | 4/26 (15.4%) |
|  | Same building | 69/131 (52.7%) | 122/298 (40.9%) | 145/319 (45.5%) | 60/135 (44.4%) | 26/59 (44.1%) | 10/25 (40.0%) | 9/26 (34.6%) |
|  | Different building | 8/131 (6.1%) | 47/298 (15.8%) | 52/319 (16.3%) | 26/135 (19.3%) | 10/59 (16.9%) | 4/25 (16.0%) | 8/26 (30.8%) |
|  | Different hospital | 6/131 (4.6%) | 9/298 (3.0%) | 8/319 (2.5%) | 0/135 (0.0%) | 0/59 (0.0%) | 0/25 (0.0%) | 0/26 (0.0%) |
|  | Not available | 36/131 (27.5%) | 77/298 (25.8%) | 62/319 (19.4%) | 28/135 (20.7%) | 18/59 (30.5%) | 6/25 (24.0%) | 5/26 (19.2%) |
| **Thromboelastometry (TEG/ROTEM)** | Same ward/adjoining | 4/131 (3.1%) | 23/298 (7.7%) | 50/319 (15.7%) | 37/135 (27.4%) | 20/59 (33.9%) | 13/25 (52.0%) | 9/26 (34.6%) |
|  | Same building | 14/131 (10.7%) | 60/298 (20.1%) | 82/319 (25.7%) | 28/135 (20.7%) | 18/59 (30.5%) | 6/25 (24.0%) | 6/26 (23.1%) |
|  | Different building | 4/131 (3.1%) | 22/298 (7.4%) | 40/319 (12.5%) | 23/135 (17.0%) | 7/59 (11.9%) | 0/25 (0.0%) | 5/26 (19.2%) |
|  | Different hospital | 22/131 (16.8%) | 60/298 (20.1%) | 40/319 (12.5%) | 9/135 (6.7%) | 2/59 (3.4%) | 2/25 (8.0%) | 1/26 (3.8%) |
|  | Not available | 87/131 (66.4%) | 133/298 (44.6%) | 107/319 (33.5%) | 38/135 (28.1%) | 12/59 (20.3%) | 4/25 (16.0%) | 5/26 (19.2%) |
| **Diagnostic imaging** | | | | | | | | |
| **Computed Tomography** | Same ward/adjoining | 7/131 (5.3%) | 24/298 (8.1%) | 26/319 (8.2%) | 5/135 (3.7%) | 2/59 (3.4%) | 1/25 (4.0%) | 2/26 (7.7%) |
|  | Same building | 65/131 (49.6%) | 187/298 (62.8%) | 181/319 (56.7%) | 61/135 (45.2%) | 17/59 (28.8%) | 11/25 (44.0%) | 5/26 (19.2%) |
|  | Different building | 15/131 (11.5%) | 61/298 (20.5%) | 73/319 (22.9%) | 46/135 (34.1%) | 25/59 (42.4%) | 3/25 (12.0%) | 12/26 (46.2%) |
|  | Different hospital | 17/131 (13.0%) | 19/298 (6.4%) | 29/319 (9.1%) | 17/135 (12.6%) | 14/59 (23.7%) | 8/25 (32.0%) | 7/26 (26.9%) |
|  | Not available | 27/131 (20.6%) | 7/298 (2.3%) | 10/319 (3.1%) | 6/135 (4.4%) | 1/59 (1.7%) | 2/25 (8.0%) | 0/26 (0.0%) |
| **Magnetic Resonance Imaging** | Same ward/adjoining | 3/131 (2.3%) | 20/298 (6.7%) | 22/319 (6.9%) | 4/135 (3.0%) | 2/59 (3.4%) | 2/25 (8.0%) | 1/26 (3.8%) |
|  | Same building | 29/131 (22.1%) | 144/298 (48.3%) | 165/319 (51.7%) | 57/135 (42.2%) | 18/59 (30.5%) | 11/25 (44.0%) | 7/26 (26.9%) |
|  | Different building | 8/131 (6.1%) | 57/298 (19.1%) | 77/319 (24.1%) | 49/135 (36.3%) | 24/59 (40.7%) | 3/25 (12.0%) | 10/26 (38.5%) |
|  | Different hospital | 31/131 (23.7%) | 47/298 (15.8%) | 36/319 (11.3%) | 19/135 (14.1%) | 14/59 (23.7%) | 7/25 (28.0%) | 7/26 (26.9%) |
|  | Not available | 60/131 (45.8%) | 30/298 (10.1%) | 19/319 (6.0%) | 6/135 (4.4%) | 1/59 (1.7%) | 2/25 (8.0%) | 1/26 (3.8%) |
| **Ultrasound imaging equipment** | Same ward/adjoining | 62/131 (47.3%) | 236/298 (79.2%) | 249/319 (78.1%) | 100/135 (74.1%) | 41/59 (69.5%) | 19/25 (76.0%) | 21/26 (80.8%) |
|  | Same building | 61/131 (46.6%) | 57/298 (19.1%) | 68/319 (21.3%) | 33/135 (24.4%) | 17/59 (28.8%) | 6/25 (24.0%) | 5/26 (19.2%) |
|  | Different building | 6/131 (4.6%) | 3/298 (1.0%) | 2/319 (0.6%) | 2/135 (1.5%) | 1/59 (1.7%) | 0/25 (0.0%) | 0/26 (0.0%) |
|  | Different hospital | 0/131 (0.0%) | 0/298 (0.0%) | 0/319 (0.0%) | 0/135 (0.0%) | 0/59 (0.0%) | 0/25 (0.0%) | 0/26 (0.0%) |
|  | Not available | 2/131 (1.5%) | 2/298 (0.7%) | 0/319 (0.0%) | 0/135 (0.0%) | 0/59 (0.0%) | 0/25 (0.0%) | 0/26 (0.0%) |
| **Facilities** | | | | | | | | |
| **Dedicated operating room for obstetric patients** | Same ward/adjoining | 46/131 (35.1%) | 197/298 (66.1%) | 215/319 (67.4%) | 97/135 (71.9%) | 39/59 (66.1%) | 17/25 (68.0%) | 20/26 (76.9%) |
|  | Same building | 59/131 (45.0%) | 88/298 (29.5%) | 92/319 (28.8%) | 35/135 (25.9%) | 18/59 (30.5%) | 8/25 (32.0%) | 6/26 (23.1%) |
|  | Different building | 6/131 (4.6%) | 2/298 (0.7%) | 6/319 (1.9%) | 1/135 (0.7%) | 2/59 (3.4%) | 0/25 (0.0%) | 0/26 (0.0%) |
|  | Different hospital | 4/131 (3.1%) | 0/298 (0.0%) | 0/319 (0.0%) | 1/135 (0.7%) | 0/59 (0.0%) | 0/25 (0.0%) | 0/26 (0.0%) |
|  | Not available | 16/131 (12.2%) | 11/298 (3.7%) | 6/319 (1.9%) | 1/135 (0.7%) | 0/59 (0.0%) | 0/25 (0.0%) | 0/26 (0.0%) |
| **Neonatal Intensive Care** | Same ward/adjoining | 20/131 (15.3%) | 72/298 (24.2%) | 117/319 (36.7%) | 55/135 (40.7%) | 19/59 (32.2%) | 9/25 (36.0%) | 9/26 (34.6%) |
|  | Same building | 27/131 (20.6%) | 90/298 (30.2%) | 148/319 (46.4%) | 67/135 (49.6%) | 33/59 (55.9%) | 16/25 (64.0%) | 16/26 (61.5%) |
|  | Different building | 2/131 (1.5%) | 7/298 (2.3%) | 6/319 (1.9%) | 3/135 (2.2%) | 6/59 (10.2%) | 0/25 (0.0%) | 1/26 (3.8%) |
|  | Different hospital | 37/131 (28.2%) | 98/298 (32.9%) | 37/319 (11.6%) | 7/135 (5.2%) | 1/59 (1.7%) | 0/25 (0.0%) | 0/26 (0.0%) |
|  | Not available | 45/131 (34.4%) | 31/298 (10.4%) | 11/319 (3.4%) | 3/135 (2.2%) | 0/59 (0.0%) | 0/25 (0.0%) | 0/26 (0.0%) |
| **Ability to accommodate COVID-19 suspected or confirmed patients** | Same ward/adjoining | 21/131 (16.0%) | 109/298 (36.6%) | 130/319 (40.8%) | 72/135 (53.3%) | 24/59 (40.7%) | 8/25 (32.0%) | 16/26 (61.5%) |
|  | Same building | 48/131 (36.6%) | 121/298 (40.6%) | 126/319 (39.5%) | 39/135 (28.9%) | 15/59 (25.4%) | 9/25 (36.0%) | 6/26 (23.1%) |
|  | Different building | 16/131 (12.2%) | 17/298 (5.7%) | 22/319 (6.9%) | 10/135 (7.4%) | 11/59 (18.6%) | 0/25 (0.0%) | 3/26 (11.5%) |
|  | Different hospital | 29/131 (22.1%) | 33/298 (11.1%) | 27/319 (8.5%) | 10/135 (7.4%) | 9/59 (15.3%) | 4/25 (16.0%) | 1/26 (3.8%) |
|  | Not available | 17/131 (13.0%) | 18/298 (6.0%) | 14/319 (4.4%) | 4/135 (3.0%) | 0/59 (0.0%) | 4/25 (16.0%) | 0/26 (0.0%) |
| **Other Specialized Clinical and Diagnostic Services** | | | | | | | | |
| **Specialist Acute Stroke Service** | Same ward/adjoining | 5/131 (3.8%) | 9/298 (3.0%) | 14/319 (4.4%) | 3/135 (2.2%) | 1/59 (1.7%) | 1/25 (4.0%) | 0/26 (0.0%) |
|  | Same building | 33/131 (25.2%) | 95/298 (31.9%) | 118/319 (37.0%) | 37/135 (27.4%) | 8/59 (13.6%) | 4/25 (16.0%) | 5/26 (19.2%) |
|  | Different building | 21/131 (16.0%) | 46/298 (15.4%) | 79/319 (24.8%) | 46/135 (34.1%) | 24/59 (40.7%) | 4/25 (16.0%) | 13/26 (50.0%) |
|  | Different hospital | 37/131 (28.2%) | 104/298 (34.9%) | 84/319 (26.3%) | 45/135 (33.3%) | 22/59 (37.3%) | 10/25 (40.0%) | 7/26 (26.9%) |
|  | Not available | 35/131 (26.7%) | 44/298 (14.8%) | 24/319 (7.5%) | 4/135 (3.0%) | 4/59 (6.8%) | 6/25 (24.0%) | 1/26 (3.8%) |
| **Specialist Burns Service** | Same ward/adjoining | 0/131 (0.0%) | 1/298 (0.3%) | 1/319 (0.3%) | 2/135 (1.5%) | 0/59 (0.0%) | 0/25 (0.0%) | 0/26 (0.0%) |
|  | Same building | 10/131 (7.6%) | 12/298 (4.0%) | 17/319 (5.3%) | 5/135 (3.7%) | 5/59 (8.5%) | 1/25 (4.0%) | 3/26 (11.5%) |
|  | Different building | 5/131 (3.8%) | 8/298 (2.7%) | 37/319 (11.6%) | 14/135 (10.4%) | 16/59 (27.1%) | 1/25 (4.0%) | 9/26 (34.6%) |
|  | Different hospital | 49/131 (37.4%) | 167/298 (56.0%) | 157/319 (49.2%) | 79/135 (58.5%) | 33/59 (55.9%) | 13/25 (52.0%) | 12/26 (46.2%) |
|  | Not available | 67/131 (51.1%) | 110/298 (36.9%) | 107/319 (33.5%) | 35/135 (25.9%) | 5/59 (8.5%) | 10/25 (40.0%) | 2/26 (7.7%) |
| **Specialist Cardiothoracic Service** | Same ward/adjoining | 0/131 (0.0%) | 3/298 (1.0%) | 6/319 (1.9%) | 2/135 (1.5%) | 1/59 (1.7%) | 1/25 (4.0%) | 0/26 (0.0%) |
|  | Same building | 5/131 (3.8%) | 24/298 (8.1%) | 55/319 (17.2%) | 18/135 (13.3%) | 5/59 (8.5%) | 4/25 (16.0%) | 5/26 (19.2%) |
|  | Different building | 5/131 (3.8%) | 13/298 (4.4%) | 52/319 (16.3%) | 28/135 (20.7%) | 15/59 (25.4%) | 2/25 (8.0%) | 7/26 (26.9%) |
|  | Different hospital | 48/131 (36.6%) | 172/298 (57.7%) | 147/319 (46.1%) | 69/135 (51.1%) | 33/59 (55.9%) | 14/25 (56.0%) | 12/26 (46.2%) |
|  | Not available | 73/131 (55.7%) | 86/298 (28.9%) | 59/319 (18.5%) | 18/135 (13.3%) | 5/59 (8.5%) | 4/25 (16.0%) | 2/26 (7.7%) |
| **Specialist Neurosurgical Service** | Same ward/adjoining | 0/131 (0.0%) | 4/298 (1.3%) | 12/319 (3.8%) | 2/135 (1.5%) | 1/59 (1.7%) | 1/25 (4.0%) | 0/26 (0.0%) |
|  | Same building | 12/131 (9.2%) | 44/298 (14.8%) | 64/319 (20.1%) | 21/135 (15.6%) | 5/59 (8.5%) | 4/25 (16.0%) | 3/26 (11.5%) |
|  | Different building | 6/131 (4.6%) | 23/298 (7.7%) | 56/319 (17.6%) | 37/135 (27.4%) | 17/59 (28.8%) | 2/25 (8.0%) | 9/26 (34.6%) |
|  | Different hospital | 51/131 (38.9%) | 155/298 (52.0%) | 133/319 (41.7%) | 63/135 (46.7%) | 32/59 (54.2%) | 12/25 (48.0%) | 13/26 (50.0%) |
|  | Not available | 62/131 (47.3%) | 72/298 (24.2%) | 54/319 (16.9%) | 12/135 (8.9%) | 4/59 (6.8%) | 6/25 (24.0%) | 1/26 (3.8%) |
| **Specialist Psychiatric Service** | Same ward/adjoining | 6/131 (4.6%) | 11/298 (3.7%) | 11/319 (3.4%) | 2/135 (1.5%) | 1/59 (1.7%) | 1/25 (4.0%) | 2/26 (7.7%) |
|  | Same building | 30/131 (22.9%) | 68/298 (22.8%) | 80/319 (25.1%) | 24/135 (17.8%) | 9/59 (15.3%) | 6/25 (24.0%) | 7/26 (26.9%) |
|  | Different building | 29/131 (22.1%) | 68/298 (22.8%) | 98/319 (30.7%) | 49/135 (36.3%) | 19/59 (32.2%) | 3/25 (12.0%) | 8/26 (30.8%) |
|  | Different hospital | 39/131 (29.8%) | 119/298 (39.9%) | 108/319 (33.9%) | 53/135 (39.3%) | 27/59 (45.8%) | 9/25 (36.0%) | 8/26 (30.8%) |
|  | Not available | 27/131 (20.6%) | 32/298 (10.7%) | 22/319 (6.9%) | 7/135 (5.2%) | 3/59 (5.1%) | 6/25 (24.0%) | 1/26 (3.8%) |
| **Specialist Trauma Service** | Same ward/adjoining | 9/131 (6.9%) | 15/298 (5.0%) | 14/319 (4.4%) | 5/135 (3.7%) | 1/59 (1.7%) | 1/25 (4.0%) | 1/26 (3.8%) |
|  | Same building | 49/131 (37.4%) | 117/298 (39.3%) | 116/319 (36.4%) | 32/135 (23.7%) | 7/59 (11.9%) | 5/25 (20.0%) | 3/26 (11.5%) |
|  | Different building | 13/131 (9.9%) | 41/298 (13.8%) | 79/319 (24.8%) | 39/135 (28.9%) | 17/59 (28.8%) | 3/25 (12.0%) | 7/26 (26.9%) |
|  | Different hospital | 29/131 (22.1%) | 84/298 (28.2%) | 81/319 (25.4%) | 51/135 (37.8%) | 32/59 (54.2%) | 10/25 (40.0%) | 14/26 (53.8%) |
|  | Not available | 31/131 (23.7%) | 41/298 (13.8%) | 29/319 (9.1%) | 8/135 (5.9%) | 2/59 (3.4%) | 6/25 (24.0%) | 1/26 (3.8%) |
| **Laboratory Services** | Same ward/adjoining | 19/131 (14.5%) | 57/298 (19.1%) | 53/319 (16.6%) | 30/135 (22.2%) | 8/59 (13.6%) | 7/25 (28.0%) | 7/26 (26.9%) |
|  | Same building | 81/131 (61.8%) | 170/298 (57.0%) | 185/319 (58.0%) | 70/135 (51.9%) | 31/59 (52.5%) | 15/25 (60.0%) | 10/26 (38.5%) |
|  | Different building | 28/131 (21.4%) | 66/298 (22.1%) | 76/319 (23.8%) | 34/135 (25.2%) | 19/59 (32.2%) | 3/25 (12.0%) | 9/26 (34.6%) |
|  | Different hospital | 2/131 (1.5%) | 5/298 (1.7%) | 5/319 (1.6%) | 1/135 (0.7%) | 1/59 (1.7%) | 0/25 (0.0%) | 0/26 (0.0%) |
|  | Not available | 1/131 (0.8%) | 0/298 (0.0%) | 0/319 (0.0%) | 0/135 (0.0%) | 0/59 (0.0%) | 0/25 (0.0%) | 0/26 (0.0%) |
| **Interventional Radiology Service (24/7/365)** | Same ward/adjoining | 4/131 (3.1%) | 16/298 (5.4%) | 23/319 (7.2%) | 11/135 (8.1%) | 3/59 (5.1%) | 4/25 (16.0%) | 2/26 (7.7%) |
|  | Same building | 36/131 (27.5%) | 79/298 (26.5%) | 118/319 (37.0%) | 51/135 (37.8%) | 24/59 (40.7%) | 7/25 (28.0%) | 7/26 (26.9%) |
|  | Different building | 15/131 (11.5%) | 36/298 (12.1%) | 63/319 (19.7%) | 29/135 (21.5%) | 17/59 (28.8%) | 4/25 (16.0%) | 8/26 (30.8%) |
|  | Different hospital | 26/131 (19.8%) | 110/298 (36.9%) | 73/319 (22.9%) | 32/135 (23.7%) | 11/59 (18.6%) | 5/25 (20.0%) | 8/26 (30.8%) |
|  | Not available | 50/131 (38.2%) | 57/298 (19.1%) | 42/319 (13.2%) | 12/135 (8.9%) | 4/59 (6.8%) | 5/25 (20.0%) | 1/26 (3.8%) |

*Data are n/N (%). Annual-delivery-volume denominators for non-Türkiye were 131, 298, 319, 135, 59, 25 and 26 units, respectively. The non-Türkiye comparator was derived by subtracting the 140 Turkish units from the corresponding totals for the full 1,133-unit MaCriCare cohort. ECMO, extracorporeal membrane oxygenation; POC, point-of-care; INR, international normalised ratio; TEG, thromboelastography; ROTEM, rotational thromboelastometry.*
