# Supplementary material for: Maternal critical care infrastructure and organisational preparedness in Türkiye: a national analysis of the MaCriCare cross-sectional survey
Source: Ann Med. 2026 Oct 10;58(1):2742563. doi: 10.1080/07853890.2026.2742563 (PMC13656688; doi:10.1080/07853890.2026.2742563)
Supplement: Supplementary file 2 [file IANN_A_2742563_SM5663.docx]

**Supplementary Table S2. Support and follow-up for Critical Care Unit patients and staff by annual delivery volume**

**Panel A. Türkiye**

| **Provision** | **1–500** | **501–1,500** | **1,501–3,000** | **3,001–4,500** | **4,501–6,000** | **6,001–7,500** | **>7,500** |
| --- | --- | --- | --- | --- | --- | --- | --- |
| Breast feeding support/initiation | 13/13 (100.0%) | 36/39 (92.3%) | 31/35 (88.6%) | 8/9 | 5/5 | 3/3 | 6/6 |
| Physical contact between mother and baby is routinely facilitated while mother is in ICU | 10/13 (76.9%) | 24/39 (61.5%) | 26/35 (74.3%) | 6/9 | 3/5 | 2/3 | 3/6 |
| Virtual contact between mother and baby is routinely facilitated while mother is in ICU (e.g. using video-calls) | 10/13 (76.9%) | 19/39 (48.7%) | 21/35 (60.0%) | 5/9 | 1/5 | 2/3 | 3/6 |
| Maternity patients are routinely given separate accommodation ('side rooms') | 8/13 (61.5%) | 26/39 (66.7%) | 18/35 (51.4%) | 6/9 | 3/5 | 3/3 | 2/6 |
| Maternity patients are routinely followed up after discharge home in a critical care clinic or equivalent | 12/13 (92.3%) | 21/39 (53.8%) | 18/35 (51.4%) | 3/9 | 2/5 | 3/3 | 3/6 |
| Maternity patients are routinely screened for psychological trauma either before or after discharge home | 7/13 (53.8%) | 6/39 (15.4%) | 10/35 (28.6%) | 1/9 | 1/5 | 1/3 | 1/6 |
| Debriefing/ psychological support for staff is available in the event of poor maternal outcome | 10/13 (76.9%) | 19/39 (48.7%) | 19/35 (54.3%) | 5/9 | 1/5 | 2/3 | 3/6 |

*Data are units reporting that the provision was available, n/N (%), except Turkish strata containing fewer than 10 units, which are shown as n/N. Denominators were units reporting access to an ICU within the same hospital in each annual delivery-volume category.*

**Panel B. Non-Türkiye**

| **Provision** | **1–500** | **501–1,500** | **1,501–3,000** | **3,001–4,500** | **4,501–6,000** | **6,001–7,500** | **>7,500** |
| --- | --- | --- | --- | --- | --- | --- | --- |
| Breast feeding support/initiation | 62/79 (78.5%) | 194/236 (82.2%) | 230/284 (81.0%) | 100/125 (80.0%) | 42/54 (77.8%) | 15/19 (78.9%) | 18/21 (85.7%) |
| Physical contact between mother and baby is routinely facilitated while mother is in ICU | 43/79 (54.4%) | 159/236 (67.4%) | 189/284 (66.5%) | 87/125 (69.6%) | 29/54 (53.7%) | 10/19 (52.6%) | 11/21 (52.4%) |
| Virtual contact between mother and baby is routinely facilitated while mother is in ICU (e.g. using video-calls) | 29/79 (36.7%) | 86/236 (36.4%) | 123/284 (43.3%) | 56/125 (44.8%) | 23/54 (42.6%) | 7/19 (36.8%) | 9/21 (42.9%) |
| Maternity patients are routinely given separate accommodation ('side rooms') | 31/79 (39.2%) | 104/236 (44.1%) | 146/284 (51.4%) | 65/125 (52.0%) | 30/54 (55.6%) | 12/19 (63.2%) | 10/21 (47.6%) |
| Maternity patients are routinely followed up after discharge home in a critical care clinic or equivalent | 15/79 (19.0%) | 56/236 (23.7%) | 99/284 (34.9%) | 47/125 (37.6%) | 19/54 (35.2%) | 3/19 (15.8%) | 6/21 (28.6%) |
| Maternity patients are routinely screened for psychological trauma either before or after discharge home | 17/79 (21.5%) | 55/236 (23.3%) | 104/284 (36.6%) | 45/125 (36.0%) | 20/54 (37.0%) | 7/19 (36.8%) | 9/21 (42.9%) |
| Debriefing/ psychological support for staff is available in the event of poor maternal outcome | 27/79 (34.2%) | 129/236 (54.7%) | 196/284 (69.0%) | 99/125 (79.2%) | 34/54 (63.0%) | 13/19 (68.4%) | 16/21 (76.2%) |

*Data are units reporting that the provision was available, n/N (%). Annual-delivery-volume denominators for non-Türkiye were 79, 236, 284, 125, 54, 19 and 21 units, respectively. The non-Türkiye comparator was derived by subtracting the eligible Turkish units from the corresponding MaCriCare support-module totals.*
